# Challenging glaucoma with emerging therapies: an overview of advancements against the silent thief of sight

**DOI:** 10.3389/fmed.2025.1527319

**Published:** 2025-03-26

**Authors:** Solange Sarkis, Chloé Chamard, Bianca Johansen, Vincent Daien, Frederic Michon

**Affiliations:** ^1^Institute for Neurosciences of Montpellier, Univ Montpellier, Institut national de la santé et de la recherche médicale (INSERM), Montpellier, France; ^2^Laboratoires Théa, Clermont-Ferrand, France; ^3^Department of Ophthalmology, Gui de Chauliac Hospital, Montpellier, France; ^4^Sydney Medical School, The Save Sight Institute, The University of Sydney, Sydney, NSW, Australia

**Keywords:** glaucoma, intraocular pressure, neuroprotection, innovative treatments, biotechnological therapies

## Abstract

Glaucoma, a leading cause of irreversible blindness, represents a significant challenge in ophthalmology. This review examines recent advancements in glaucoma treatment, focusing on innovative medications and creative strategies. While new agents offer promising methods for lowering intraocular pressure (IOP), they also pose challenges related to efficacy and side effects. Alongside IOP reduction, emerging neuroprotective approaches are being explored to safeguard retinal ganglion cells (RGCs) from glaucoma-induced damage. The review also evaluates the potential of novel drug delivery systems, such as biodegradable implants and nanoparticles, to enhance treatment effectiveness and patient adherence. Additionally, it highlights the role of personalized medicine in identifying new biomarkers and customizing therapies based on individual genetic and environmental factors.

## 1 The pathophysiology of glaucoma

**Figure 1:**
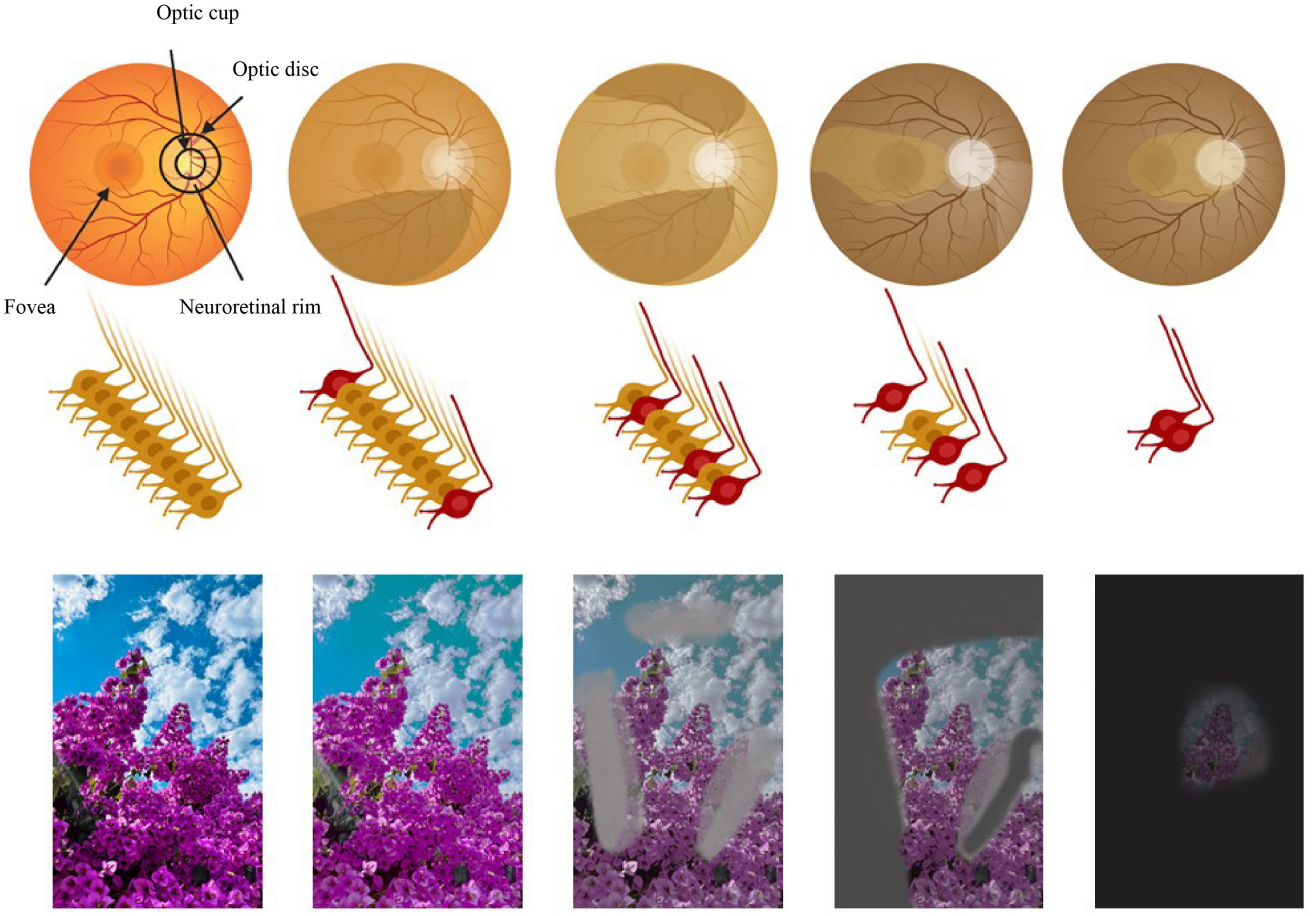
The path of vision loss [adapted and modified from Nagstrup et al. (321)]. Row 1: the retina. Row 2: the optic nerve and the retinal ganglion cells. Row 3: the perceived image. Columns 1 to 5 illustrate the progression of glaucoma: column 1 shows a normal eye and column 5 the final stage of glaucoma. Figure designed partly with Biorender, https://app.biorender.com/illustrations.

Glaucoma is a group of chronic, progressive ocular neuropathies characterized by structural changes to the optic nerve rim and retinal nerve fiber layer (RNFL) loss, leading to related visual field defects, and eventual blindness (1, 2). Crucial neurons integral to the central nervous system possess axons extending into the optic nerve, with their cell bodies residing in the inner retina. The degeneration of these neurons leads to the characteristic cupping of the optic disc and, ultimately, vision loss (1) (Figure 1).

**Figure 2:**
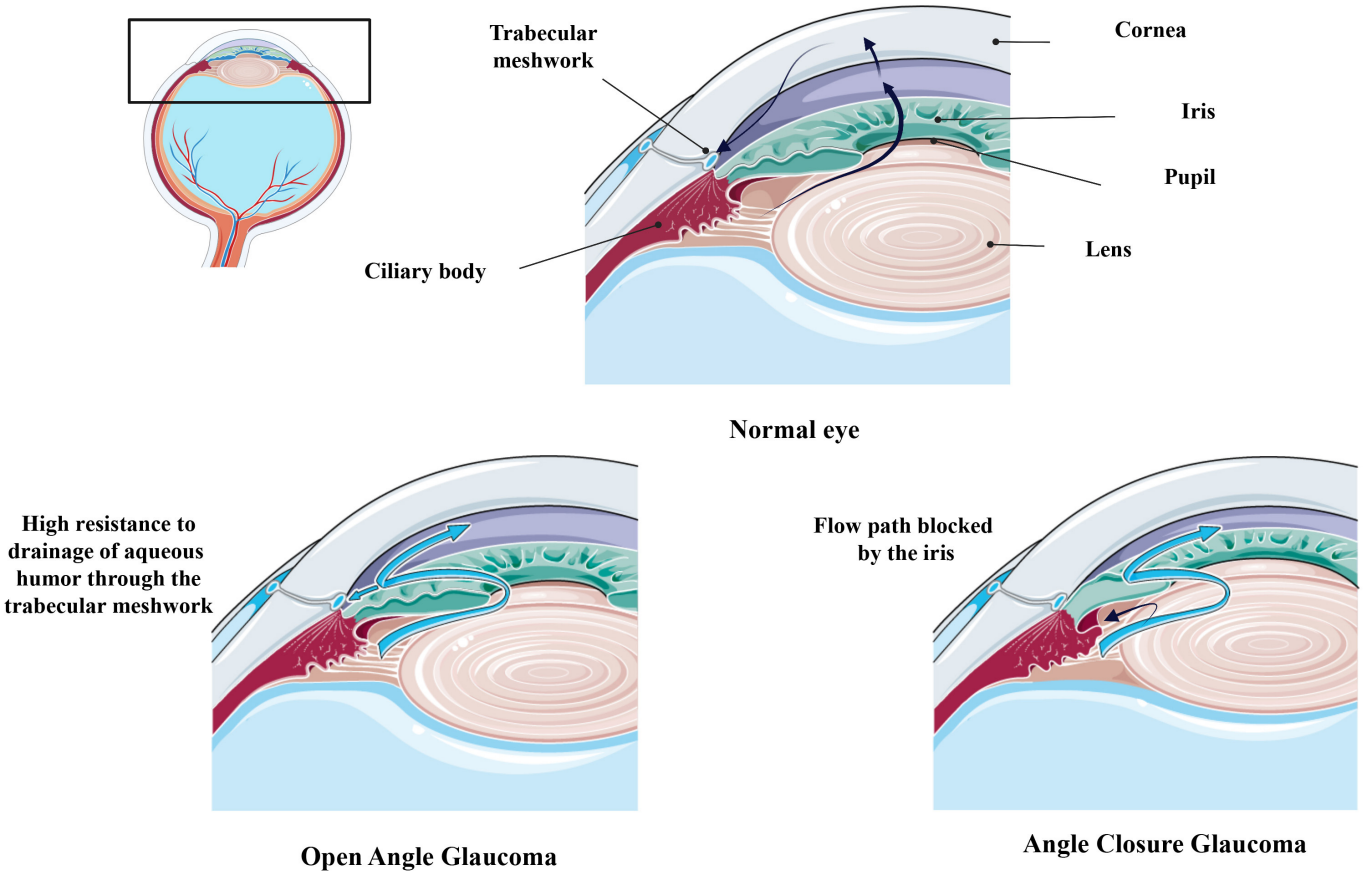
Comparing Healthy and Glaucomatous Eyes [adapted and modified from Weinreb et al. (1)]. Figure designed partly with Biorender, https://app.biorender.com/illustrations and Smart servier, https://smart.servier.com/.

Glaucoma primarily presents in two forms: open-angle glaucoma (OAG) and angle-closure glaucoma (ACG). Among the open-angle glaucomas, primary open-angle glaucoma (POAG) is the most common form, while primary angle-closure glaucoma (PACG) is the most common type of angle-closure glaucoma (2) (Figure 2). POAG arises from dysfunction of the trabecular meshwork (TM), which is responsible for draining aqueous humor (AH). This dysfunction leads to increased intraocular pressure (IOP), causing mechanical stress and eventual death of RGCs (3). POAG can be further divided into low-tension glaucoma, characterized by IOP levels below 21 mmHg, which is often associated with a higher incidence of retinal nerve fiber layer hemorrhages (4, 5). In contrast, ACG arises from contact between the iris and the TM, obstructing AH outflow. This obstruction leads to increased IOP and subsequent damage to the optic nerve (3, 6). Other forms of glaucoma include secondary open-angle glaucoma (SOAG), which can arise from eye injuries, diseases, or surgical procedures like laser treatments. SOAG may result from TM blockage due to the accumulation of inflammatory cell debris or mechanical deformation (7). Steroid-induced glaucoma is another form of secondary glaucoma, occurring due to prolonged corticosteroid use, which can lead to increased IOP and optic nerve damage (8, 9). Pseudoexfoliation syndrome (PES) is characterized by the abnormal accumulation of a gray-white, fibrillar extracellular matrix material, known as pseudoexfoliative material (PXM), in various ocular and extraocular tissues. This material is primarily deposited on the lens capsule, zonular fibers, iris, TM, and conjunctiva, leading to significant ocular manifestations, including glaucoma (10–12). Additionally, pigmentary glaucoma occurs when pigment granules from the posterior iris break away and obstruct the TM (7, 11). Low-tension glaucoma, on the other hand, is not clearly associated with IOP but rather with vascular dysfuntion, leading to optic nerve damage despite normal pressure levels (13, 14).

The global prevalence of glaucoma among individuals aged 40 to 80 years is estimated at 3.54% (15). Within this demographic, primary open-angle glaucoma (POAG) is most prevalent in Africa at 4.20%, while primary angle-closure glaucoma (PACG) is most common in Asia, with a prevalence of 1.09%. In 2013, an estimated 64.3 million people aged 40 to 80 were living with glaucoma worldwide, with projections increasing to 76.0 million by 2020 and 111.8 million by 2040 (15). According to a Bayesian meta-regression model, men are more likely to develop POAG than women. After controlling for factors such as age, gender, type of habitation, response rate, and study year, individuals of African descent showed a higher likelihood of having POAG compared to those of European descent. Additionally, urban residents were more prone to POAG than their rural counterparts (15).

Each type of glaucoma necessitates tailored treatment strategies focused on reducing IOP. Current treatments for glaucoma primarily aim to reduce IOP, which remains the only modifiable risk factor known to slow disease progression (16–18). Medications, such as prostaglandin analogs, beta-blockers, alpha agonists, and carbonic anhydrase inhibitors, are commonly prescribed to either decrease AH production or increase its outflow (19). As an alternative to eyedrops, laser therapy, like selective laser trabeculoplasty (SLT), can be utilized to enhance fluid drainage (20, 21). For more advanced or refractory cases, surgical interventions such as trabeculectomy, glaucoma drainage devices, and minimally invasive glaucoma surgeries (MIGS) provide additional options to lower IOP (22–24). While these strategies have proven effective in preserving vision, there remains an ongoing need for novel approaches that address the underlying neurodegeneration associated with glaucoma and improve patient outcomes.

As advancements in understanding the pathophysiology of glaucoma continue, innovative therapeutic approaches are emerging to challenge the limitations of conventional treatments. Novel pharmacological agents targeting neuroprotection, minimally invasive surgical techniques, and cutting-edge technologies like gene therapy and regenerative medicine hold the promise of reshaping the management of glaucoma. This review aims to explore these pioneering strategies, highlighting their potential to not only lower IOP but also preserve and restore vision, ultimately offering new hope for patients facing this progressive optic neuropathy.

## 2 Advances in glaucoma therapy

### 2.1 IOP-focused medications

#### 2.1.1 Rho-kinase inhibitors

Rho-kinase is a protein kinase that functions as serine/threonine kinase, regulating calcium-dependent smooth muscle contraction and cytoskeletal dynamics. It plays a critical role in modulating AH outflow, cell adhesion, actomyosin contraction, and cellular rigidity (19). Recently, Rho-kinase (ROCK) inhibitors, such as Ripasudil (K-115) and Netarsudil (AR-13324), have been approved for glaucoma treatment, offering the dual benefits of lowering IOP and providing neuroprotection. These agents modify the cytoskeletons of the TM and Schlemm's canal (SC), which reduces outflow capacity through the conventional route and consequently lowers IOP. By inhibiting RhoA GTPase signaling, these agents induce relaxation of TM cells, resulting in increased AH outflow (25). This mechanism is particularly crucial in the context of ocular hypertension (OHT), where high resistance to AH outflow plays a key role in disease progression. Research has demonstrated that ROCK inhibitors can profoundly modulate the contractile properties of TM tissue, enhancing its ability to facilitate efficient AH drainage and potentially mitigating the pathophysiological burden associated with increased IOP (26). However, because the IOP-lowering effect of ROCK inhibitors is less potent compared to prostaglandin analogs (PGAs), they are primarily used as adjunctive therapies (19, 27).

Ripasudil was first approved in Japan in 2014 and has undergone several clinical trials led by Tanihara et al. that confirmed its efficacy in reducing IOP, particularly with a 0.4% concentration administered twice daily (28, 29). Long-term surveillance has shown no significant safety concerns, although common side effects include conjunctival hyperemia, minor microhemorrhages near the limbus, and blepharitis (27, 30, 31). Ripasudil has also been studied in combination with other treatments, demonstrating effective IOP reduction across various glaucoma types, including secondary glaucomas (32–35). In contrast, netarsudil received FDA approval in 2017 and is characterized as both a ROCK and norepinephrine transporter inhibitor, with substantial IOP-lowering effects observed in clinical trials (36). However, it is considered slightly less effective than latanoprost and timolol when used alone (37). The combination of netarsudil with latanoprost showed superior efficacy in reducing IOP compared to monotherapy (38). Other ROCK inhibitors, such as SNJ-1656, AR-12286, PHP-201, and ITRI-E-212, have shown considerable promise in glaucoma treatment and have been evaluated in early clinical trials (39–42).

Additionally, several clinical trials for other ROCK inhibitors have been registered. These include INS115644, which was a Phase 1 trial for POAG and OHT in 2007, and INS117548, another Phase 1 trial for bilateral OHT or early POAG in 2008 (36). LX7101 has undergone both Phase 1 and 2 trials for POAG and OHT starting in 2012, while ATS907 was assessed in Phase 1 and 2a trials in the same year (36). A Phase 2 trial for ATS907 also took place in 2012 (36). More recently, H-1337 was evaluated in a Phase 2 trial for POAG and OHT in 2023 (36). Overall, while several ROCK inhibitors are undergoing clinical trials or are in preclinical stages, their safety, efficacy, and potential combinations in treating glaucoma continue to be areas of active research, showing promise for improving treatment outcomes in patients with this condition.

#### 2.1.2 Nitric oxide-donating prostaglandin analog

In 2017, the FDA approved latanoprostene bunod, a nitric oxide-donating prostaglandin F2α receptor agonist, for the treatment of OAG and OHT by reducing IOP. Its mechanism involves enhancing the outflow of AH through SC, the TM, and the uveoscleral pathway. The release of nitric oxide activates the guanylate cyclase-1-cGMP pathway, which promotes relaxation of the TM and facilitates increased AH outflow (43). Clinical studies comparing latanoprostene bunod administered once daily with timolol administered twice daily demonstrated that latanoprostene bunod significantly lowered IOP at all assessed time points. Patients using latanoprostene bunod had a higher percentage of individuals with a mean IOP ≤ 18 mmHg and a reduction in IOP ≥25% compared to those using timolol. Additionally, patients transitioning from timolol to latanoprostene bunod experienced a sustained decrease in their mean diurnal IOP, although the incidence of side effects was higher with latanoprostene bunod compared to timolol (44, 45). Building on these findings, a double-masked, placebo-controlled Phase 3b clinical trial (NCT05938699) aims to investigate the effects of NCX 470 ophthalmic solution (0.1%) on AH dynamics in healthy volunteers and subjects with OAG. This study will provide further insights into the action of NCX 470 on key AH parameters and its potential as a treatment for IOP management in glaucoma patients.

#### 2.1.3 FC Rho-kinase inhibitor/latanoprost

The FDA has granted approval for the first combination treatment of a prostaglandin analog and a rho-kinase inhibitor for managing OAG and OHT. This combination consists of netarsudil 0.02% and latanoprost 0.005% in an ophthalmic solution (46). By decreasing AH production, enhancing AH through the TM, and reducing episcleral venous pressure, netarsudil effectively lowers IOP. It works synergistically with latanoprost, which enhances uveoscleral outflow, further reducing IOP. Comparative clinical studies have demonstrated that the fixed combination of netarsudil/latanoprost significantly and consistently outperforms either drug used alone in lowering IOP (38, 47). A Phase 3 study (NCT03284853) and phase 2 study (NCT02057575) comparing PG324 (Netarsudil/Latanoprost) to Bimatoprost/Timolol and AR-13324, respectively, in glaucoma patients found both treatments equally effective in reducing IOP. However, PG324 had a better safety profile with fewer adverse effects, making it a promising alternative for managing glaucoma and ocular hypertension. Similarly, 12-month study with a 2-month extension (NCT02558400) and a separate 3-month study (NCT02674854) compared PG324 to AR-13324 and Latanoprost in patients with elevated IOP. Both confirmed the superior efficacy PG324 in sustained IOP reduction over individual treatments, with the longer study providing additional long-term safety data. Switching to Netarsudil/Latanoprost from various latanoprost-based regimens effectively lowered IOP in glaucoma and ocular hypertension patients over 12 weeks, demonstrating its efficacy as a replacement therapy (NCT05283395).

Common ocular side effects include mild conjunctival hyperemia, conjunctival hemorrhage, and corneal verticillata (38, 47). New therapeutic options, such as netarsudil, a rho-kinase inhibitor, latanoprostene bunod, and the netarsudil/latanoprost fixed combination, offer diverse mechanisms of action for treating these conditions. However, these medications, containing benzalkonium chloride (BAK), may exacerbate dry eye symptoms and contribute to ocular surface diseases. Additionally, the cost of these medications can pose a significant barrier to long-term treatment adherence (19).

#### 2.1.4 Cannabinoids

Since the early 1970s, research has explored the effects of cannabinoids (CB) on glaucoma (48, 49). Receptors CB1 and CB2, which are natural targets for endocannabinoids like Anandamide (AEA), Palmitoylethanolamide (PEA), and 2-Arachidonoylglycerol (2-AG), are present in the human retina, ciliary body, and retinal pigment epithelium. These receptors interact with cannabinoids, which can affect IOP and other retinal functions. The administration of exogenous CB may influence IOP regulation, potentially through various mechanisms (19, 48). Control of IOP is influenced by CB1 receptors found in the SC, TM, iris, ciliary body muscle, and ciliary pigmented epithelium. Additionally, CB1 receptors may modulate prostanoid synthesis via cyclooxygenase-2 (COX-2) regulation (19, 48). CB can lower IOP by modulating the production and outflow of AH, both trabecular and uveoscleral (48). This effect is partly achieved through the inhibition of calcium influx in presynaptic channels, which reduces noradrenaline release in the ciliary body and subsequently decreases AH production (19). The CB1 receptor interacts with AEA and synthetic cannabinoids like CP55,940 to control ciliary muscle contraction, affecting IOP. Activation of CB1 receptors in the ciliary body muscle may also induce vasodilation, further reducing AH production (48).

The tetrahydrocannabinol (THC) isomer trans-delta-9-tetrahydrocanabinol (delta-9-THC) and cannabigerol affect the dilation of SC and facilitate AH outflow, while AEA and CP55,940 act on the ciliary muscle. Moreover, AEA and delta-9-THC stimulate COX-2 expression, which increases the synthesis of metalloproteinases 1, 3, and 9, as well as prostaglandin E2 (PGE2). This leads to a decrease in IOP due to remodeling of the extracellular matrix. Despite promising results from several studies, further clinical trials are necessary to fully understand the precise role of these CB in the physiological regulation of IOP (48). While cannabinoids show potential benefits for glaucoma patients, including reduced IOP, several issues remain unresolved. Research highlights challenges such as irregular absorption and variable efficacy of oral cannabinoid use. Topical administration shows some promise but requires further investigation to confirm its safety and effectiveness (50). One strategy to enhance THC ocular bioavailability involves developing a hydrophilic prodrug, Δ9-tetrahydrocannabinol-valine-hemisuccinate. When delivered via a lipid-based nanoparticulate vector, this prodrug has demonstrated significant IOP reduction (by 30% from baseline) in animal studies, with effects lasting up to 6 h (51). However, more research is needed to validate these findings in human clinical trials before considering this as a viable glaucoma treatment option. Additionally, cannabinoids may possess antioxidant, anti-inflammatory, and neuroprotective properties that could protect RGCs from glaucoma-induced damage (52). A clinical study on glaucoma patients demonstrated that PEA supplementation improved RGCs function, as assessed by pattern electroretinogram (PERG), over 3 month period. However, no significant effects were observed on IOP, visual acuity, central corneal thickness (CCT), ganglion cell complex (GCC) on optical coherence tomography (OCT), or quality of life measures (NCT04088084). Another study aims evaluate the effects of single-dose dronabinol, a synthetic THC, on ocular blood flow in patients with POAG. It will measure changes in optic nerve head (ONH) blood flow, retinal blood flow, retinal oxygen saturation, and neurovascular coupling. Results have not been reported yet (NCT04596826).

While CB is effective in its intended purpose, it is essential to be aware of the potential side effects associated with its use. Several studies showed deleterious effects in the retina and ganglion cells (53). Lucas et al. studied the link between cannabis consumption and the phenomenon of retinal neuronal activity during a stimulus-free resting state. According to their research, there appears to be a correlation between THC use and an increase in neuronal background noise in the brain and retina. This could be an indication of the neurotoxicity of cannabis on retinal neuron dynamics, which is probably caused by changes in neurotransmitter release (53, 54). According to research by Schwitzer et al., regular cannabis users have delayed responses from their ganglion and bipolar cells, resulting in a delay in the brain processing of visual information. They were able to show that this delay in transmission could lead to changes in color vision and clarity using a multifocal electroretinogram (53, 55, 56).

Recent studies have reaffirmed that cannabinoids can reduce IOP, though with limitations such as a short duration of effect and potential side effects like euphoria and hypotension (57, 58). For example, while THC has shown the ability to lower IOP, evidence supporting its long-term efficacy and safety for glaucoma treatment remains insufficient (59).

Moreover, cannabinoid use carries risks, including toxicity, social, and cognitive disorders. It is contraindicated in individuals with serious health issues and may interact adversely with other illicit drugs (53). Despite these concerns, the regulatory environment around cannabinoids is shifting, with increasing acceptance in various regions, which has led to growing public interest in their potential for managing glaucoma (60, 61). Surveys reveal that many patients are aware of the possible benefits of medical marijuana for glaucoma, yet healthcare professionals still fail to provide consistent and clear information (60, 61). This gap underscores the need for more education and research to clarify the role of cannabinoids in glaucoma treatment and to address patient concerns (62).

While cannabinoids have shown potential, they are not currently recommended as a first-line treatment for glaucoma by major ophthalmological societies. This is mainly due to the lack of substantial clinical evidence (61, 62). To better understand the therapeutic potential of cannabinoids, further research is needed, particularly regarding their neuroprotective properties and the mechanisms through which they may relieve glaucoma symptoms (52, 63). Exploring cannabinoids as adjunct therapies alongside traditional glaucoma treatments may also offer promising opportunities for future investigations (58, 64).

#### 2.1.5 Melatonin

Melatonin, primarily known for its role in regulating sleep-wake cycles, has several other physiological functions. Notably, evidence supports its involvement in regulating IOP and AH dynamics, as melatonin receptors are found in various ocular tissues (19, 65, 66). The primary melatonin receptors, MT1, MT2, and MT3, are notably expressed in key ocular structures, including the ciliary epithelium (66, 67), the iris and retina (68). These receptors, which belong to the G-protein-coupled receptor (GPCR) family, mediate effects of melatonin through distinct signaling pathways, often involving the inhibition of cyclic AMP (69). The MT3 receptor, expressed by the sympathetic nervous system, plays a role in controlling the production and outflow of AH in the eye, with melatonin influencing these processes via cholinergic and noradrenergic pathways. This influence includes the regulation of adrenergic receptor expression, particularly the up- and down-regulation of alpha-2 and beta-2 receptors, which may contribute to the hypotensive effects of melatonin on the eyes (65).

Recent studies indicate that the absence of MT1 receptors in mice leads to increased nocturnal IOP and a decrease in RGCs, highlighting a possible connection between impaired melatonin signaling and the development of glaucoma (68). In the porcine ciliary epithelium, melatonin has been shown to stimulate AH secretion via MT3 receptors, facilitating chloride and fluid transport (67). Additionally, the presence of melatonin receptor mRNA and protein expression in the nonpigmented ciliary epithelial cells of *Xenopus laevis* provides further evidence of melatonin the direct influence of melatonin on AH secretion (70).

Melatonin treatment has been shown to significantly reduce IOP in glaucomatous animals, suggesting its potential as both a stand-alone and adjunct therapy (71, 72). Beyond its effects on IOP, melatonin also possesses neuroprotective properties that are particularly relevant for glaucoma management. It helps mitigate two key contributors to RGC degeneration: inflammation and oxidative stress (73, 74). Additionally, its strong antioxidant capacity supports retinal cellular integrity and prevents apoptotic cell death (73, 75). Preclinical studies further indicate that melatonin enhances neuroprotection by increasing GABA levels, promoting retinal glutamate clearance, and boosting antioxidant activity in animal models of glaucoma (73).

Agomelatine, a non-selective agonist of the MT1 and MT2 melatonin receptors, exerts a hypotensive effect on IOP primarily through the activation of MT2 and MT3 receptors (65). Research indicates that oral administration of the melatonin agonist agomelatine can consistently reduce IOP in POAG patients, even when used alongside various topical treatments. These findings suggest that agomelatine may be a valuable therapeutic option for POAG and support further development of agomelatine for topical use, which could potentially offer more potent and prolonged effects (65). Additionally, the presence of melatonin receptors in ocular tissues highlights the potential for targeted therapeutic interventions through topical administration, leveraging these receptors to elicit beneficial effects (76). Recent advancements in drug delivery systems, particularly nanotechnology, have further enhanced the feasibility of melatonin-based ocular treatments by improving its solubility, stability, and permeability across ocular barriers (77, 78). These innovations enable sustained release formulations, optimizing therapeutic efficacy while ensuring better patient compliance (78). Furthermore, studies indicate that melatonin levels in the AH naturally rise in response to increased IOP, suggesting an intrinsic protective mechanism. Harnessing this response through topical melatonin administration could provide a novel, physiologically relevant approach to managing ocular conditions associated with IOP elevation (79).

Moreover, melatonin also interacts with dopamine, a neurotransmitter involved in regulating circadian rhythms, mood, and behavior. By modulating dopaminergic pathways, melatonin may affect AH production (through dopamine DA1 receptors) and outflow (through dopamine DA2-3 receptors) (65). Additionally, melatonin and its agonists can reduce AH production by down-regulating carbonic anhydrase, an enzyme involved in the AH production process (80). Furthermore, melatonin-induced changes in glycosaminoglycan production may impact the TM, which regulates IOP, potentially influencing the pathophysiology of POAG (81).

Preclinical studies have demonstrated that melatonin has the potential to lower IOP and protect RGCs, making it a promising candidate for glaucoma treatment. However, its efficacy in glaucoma patients remains untested due to the absence of large-scale clinical trials, highlighting the need for further research. Further studies are necessary to better understand intra-subject variability and consider the natural fluctuations in IOP related to daily activities and circadian rhythms.

#### 2.1.6 Connective tissue growth factor

Connective tissue growth factor (CTGF) is an immediate early protein regulated by transforming growth factor-beta (TGF-β) and it plays a crucial role in the proliferation of fibroblasts and the production of the extracellular matrix (ECM) (82–84). CTGF is present in various structures of the human eye. Van Setten et al. demonstrated that in the cornea, CTGF is detected in the epithelium, particularly in the basal layers, as well as in stromal keratinocytes and endothelial cells (83). Adjacent conjunctival epithelial cells also display CTGF (83). In the iris, CTGF is observed in both the sphincter and dilator muscles, and in the vessels of the iris and ciliary body, where it is derived from vascular endothelium but not from vascular smooth muscle cells (83). Within the ciliary body, CTGF is present in the smooth muscle cells of the ciliary muscle and the non-pigmented epithelium (83). In the retina, CTGF is localized to the nerve fiber layer (NFL) and weakly in the inner and outer plexiform layers (IPL/OPL) (83). The choroid exhibits CTGF in the choriocapillaris and blood vessels, while a few cells in the optic nerve head and lamina cribrosa also display CTGF positivity (83). The wide distribution of CTGF across various ocular structures, combined with its presence in the AH means that CTGF is transported to the TM, where it may alter outflow resistance, ultimately impacting IOP homeostasis.

This broad distribution of CTGF highlights its potential role in the pathogenesis of glaucomatous optic neuropathy. Both CTGF and its upstream regulator, TGF-β, are implicated in the pathogenesis of glaucomatous optic neuropathy due to their roles in ECM remodeling. Overexpression of these growth factors is associated with glaucoma, as it disrupts ECM turnover, leading to excessive ECM accumulation in the AH outflow pathway, increasing outflow resistence and ultimately resulting in elevated IOP, which contributes to glaucoma development (83, 84).

Recent advances have introduced the use of hyaluronan-coated nanoparticles for delivering anti-CTGF small interfering RNA (siRNA) directly into the eye's anterior chamber. This method enhances penetration into the outflow regions, such as the TM and SC. Hyaluronan binding to CD44 receptors, which are highly expressed in glaucoma-affected eyes, facilitates this process. Combining RNA interference with hyaluronan-coated nanoparticles offers a promising strategy for treating glaucoma (85). Another approach involves the use of microRNA mimetics, specifically miR-18a-5p, to downregulate connective CTGF expression and inhibit TGFβ2-induced TM cell contractility, a strategy that has been proposed as a viable therapeutic option (86). Beyond its role in disease pathogenesis, CTGF has also been implicated in the fibrotic response following glaucoma filtration surgery. Research indicates that CTGF overexpression contributes to excessive collagen deposition and fibroblast proliferation, leading to postoperative scarring and surgical failure (87, 88). A deeper understanding of the molecular pathways involving CTGF could facilitate the development of antifibrotic strategies, such as microRNA-26a and the use of CRISPR-Cas9 aimed at enhancing surgical success rates and minimizing complications (87, 89). In summary, therapeutic strategies targeting CTGF, such as siRNA and microRNA-based approaches, show promise in improving glaucoma management. However, further clinical trials are needed to evaluate the efficacy and safety of these innovative treatments before they can become mainstream options for patients.

#### 2.1.7 Adenosine

Adenosine is a ubiquitous local modulator that interacts with four membrane receptors: A1, A2A, A2B, and A3 to regulate various physiological and pathological processes (90). These receptors influence AH dynamics, IOP, retinal function, blood flow, and neuroprotection (90). The ciliary processes in rat eyes express A1, A2A, and A2B receptor mRNAs, while the retina expresses A1 and A2A (91).

Activation of A1 receptors in the ciliary body and TM reduces AH production and outflow resistance, lowering IOP (90). A3 receptor antagonists lower IOP by preventing adenosine-induced activation of Cl- channels in ciliary epithelial cells, while A2A receptor activation in Schlemm's canal cells can have bidirectional effects on IOP (90, 92). In pseudoexfoliation syndrome, A3 receptor expression is upregulated in the ciliary epithelium, potentially offering cytoprotective effects (93).

Adenosine receptor signaling pathways involve molecular mechanisms such as adenylyl cyclase, phospholipase-inositol triphosphate-diacylglycerol, and phosphatidylinositol-3-kinase, which activate MAPK pathways that influence gene transcription (94). In addition to adenosine receptor agonists and antagonists, drugs that target intracellular signaling molecules—such as Ras proteins, G proteins, and the small GTPase Rho—are also being explored (94). Emerging therapies, including Rho-kinase inhibitors and adenosine receptor ligands, show promise for improving AH outflow and offering neuroprotection for RGCs (95).

Several clinical studies, including Phase 2 and 3 trials, have focused on evaluating the efficacy of various topical formulations in lowering IOP in adults with OHT or POAG, comparing them to existing therapies like latanoprost and timolol (NCT01917383, NCT02565173). These studies demonstrated that trabodenoson, anA_1_ adenosine receptor agonist, has shown significant potential in reducing IOP, with Phase 2 trials revealing a 6–7 mmHg reduction. However, Phase 3 trials encountered setbacks due to incorrect dosages and regimens. Trabodenoson, optimally dosed at 0.6%, helps control IOP through two mechanisms: slow-mode turnover of the TM and fast-mode vascular effects. Trabodenoson impact on TM rejuvenation is strategically important for addressing the progression of glaucoma, enhancing treatment responsiveness, and preventing the disease from reaching a terminal stage, such as blindness (96). When combined with latanoprost, the two therapies work synergistically, with trabodenoson rejuvenating the TM and latanoprost enhancing AH outflow. This fixed-dose combination results in significant IOP reduction and offers a favorable safety profile for patients with OHT or POAG (NCT02829996).

Safety and tolerability were key focus areas across these studies, with comprehensive physical health checks, ECGs, blood tests, and monitoring for adverse events ensuring the drug safety profiles. Additionally, some studies, such as those involving oral doses of PBF-677 in healthy volunteers, employed dose-escalation methodologies to determine the maximum tolerated dose while closely monitoring for potential side effects (NCT02639975).

Similarly, the study evaluating INO-8875 eye drops aims to assess their tolerability, safety, and effectiveness in adults with glaucoma or OHT. By focusing on the ability of INO-8875 to reduce IOP while ensuring minimal side effects, this trial seeks to establish the drug as a promising treatment option for managing these eye conditions (NCT01123785). These trials, along with ongoing studies on trabodenoson, underline the evolving landscape of glaucoma treatment and the importance of finding safe, effective therapies for IOP management.

### 2.2 Neuroprotection-focused medications

While IOP reduction remains the primary clinically validated method for controlling glaucoma and slowing disease progression, it is increasingly recognized that developing neuroprotective strategies is essential. Neuroprotection extends beyond merely lowering IOP; it also involves enhancing cell survival and bolstering resistance to optic nerve damage.

Several potential neuroprotective targets have shown promise in preclinical studies, but failed to demonstrate efficacy in clinical trials, despite their effects on IOP and/or neuroprotection (97). A particularly promising strategy would be to develop treatments that simultaneously lower IOP and provide neuroprotection. However, achieving this is challenging due to the complexity of glaucoma and the nature of optic nerve damage.

**Figure 3:**
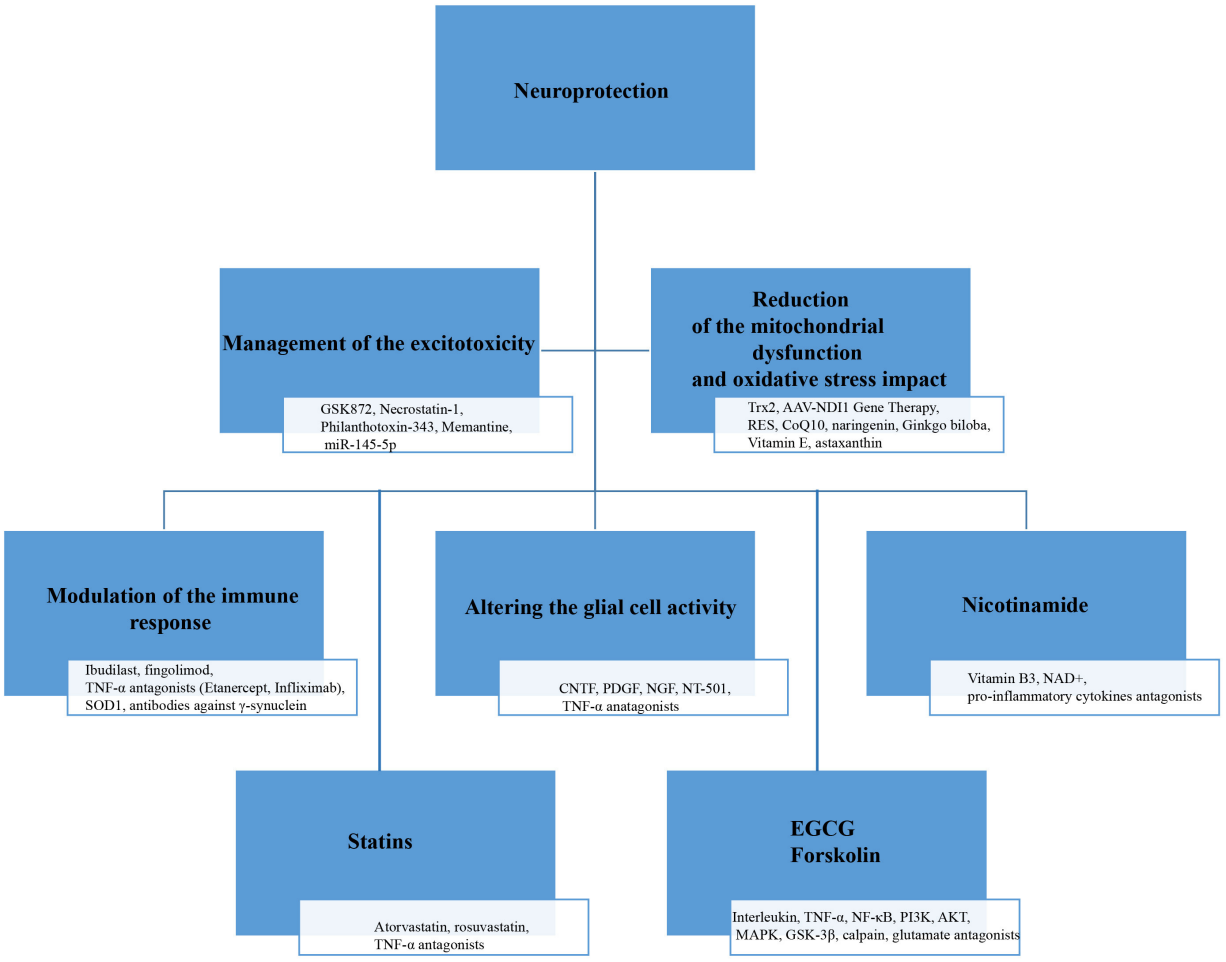
Overview of key molecules involved in neuroprotection in glaucoma.

The term “neuroprotection” in the context of glaucoma refers to methods aimed at preventing damage and degeneration of RGCs and their axons, which are key features of the disease. Treatment strategies can be categorized based on their targets, including the disruption of excitotoxicity, management of oxidative stress and mitochondrial dysfunction, treatment of inflammation and abnormal immune responses, modulation of glial cells, and the use of gene and stem cell therapies. Additionally, molecules such as nicotinamide, statins, and neurotrophic factors are being explored for their potential benefits (Figure 3). It is important to note that this classification is somewhat arbitrary, as many of these targets are interconnected and involved in multiple pathways The development of this neuroprotective drugs for glaucoma faces significant challenges, with high costs and risks. These challenges stem from an incomplete understanding of glaucomatous optic neuropathy and retinopathy, which can be divided into several areas: the mechanisms of pathogenesis remain uncertain, therapeutic targets are not clearly defined, and there is a lack of validated preclinical models (98, 99). Over the past several decades, numerous models have been developed for glaucoma research (100). *In vitro* models include twelve immortalized RGC lines, primary RGC cultures, and, more recently, RGCs derived from induced pluripotent stem cells. Inherited and induced rodent models have largely replaced the primate models used in the 1970s and 1980s (100). Most glaucoma models focus on increasing IOP, employing various methods to either elevate episcleral venous pressure or enhance aqueous outflow resistance. Additionally, glaucoma research has employed models that induce an acute rise in IOP, as well as other optic nerve injury models (101). Every glaucoma model has its strengths and limitations. Cell culture systems offer a rapid approach to evaluate potential neuroprotective agents. However, they fall short in accurately representing the complexities of the human condition. Among animal models, laser-induced IOP elevation in primates is arguably the most physiologically relevant. Yet, ethical concerns and high costs have significantly restricted its use (101). The most widely used genetic rodent model, the DBA/2J mouse, allows for large-scale studies but is also associated with various non-glaucomatous pathologies (102). Induced rodent models, while useful, exhibit varying degrees of reproducibility and inconsistencies in the extent of RGC damage they produce (101). Given these limitations, it seems clear that the best strategy for glaucoma neuroprotection research may be to focus on relatively safe neuroprotective agents that can be rapidly tested in humans, minimizing the time spent on animal studies that may not yield reliable or relevant results.

The slow progression of glaucoma, the variability in its pathogenesis, and the need for clear evidence beyond normal IOP reduction make clinical investigations into neuroprotection particularly challenging. These studies are often complex, costly, and time-consuming due to the chronic nature of glaucoma and its gradual development (103, 104). However, strategic approaches can significantly reduce the workload and sample size required to achieve conclusive results. While OCT has become a widely used clinical tool, automated perimetry has traditionally been considered the gold standard for primary outcomes in clinical glaucoma research (105). In neuroprotection studies, guided progression analysis using Cirrus HD-OCT offers a trend-based statistical approach that may replace perimetry as the primary outcome (101). A promising strategy could involve focusing on “low-hanging fruit,” where results can be evaluated more quickly. In cases of acute glaucoma or instances where IOP management may temporarily be suboptimal, randomized controlled trials comparing neuroprotectant treatment to a placebo could be particularly useful (101). Additionally, leveraging national or international databases would allow for the selection of individuals with chronic glaucoma and rapid progression, enhancing the efficiency of the research (101). Additionally, the effectiveness of neuroprotective medications relies on patients adhering to their treatment plans. The complexity of certain treatments, such as those requiring intravitreal injections or continuous monitoring, may discourage patients from consistently following their prescribed regimens (106). Moreover, the overall therapeutic burden on patients must be carefully considered when integrating neuroprotective strategies into current glaucoma management protocols. For example, while adjunctive therapies have shown promise in enhancing neuroprotection, the risks associated with non-adherence and the potential increase in medication burden should be evaluated before their inclusion in standard care (107, 108). To optimize patient adherence and achieve the most favorable outcomes, it will be essential to develop convenient delivery methods and ensure that patients are thoroughly educated about the benefits and importance of neuroprotective medications (109). Ultimately, the holy grail of laboratory glaucoma research is to develop animal models that more closely resemble human disease in order to validate new therapeutic agents before progressing to human trials. While there is an ever-increasing abundance of preclinical research, clinical translation remains in its early stages and is not without its inherent challenges. Refining clinical trial designs and utilizing validated monitoring techniques will be essential in improving the cost-efficiency and success of clinical neuroprotective trials in glaucoma research.

#### 2.2.1 Management of the excitotoxicity (NMDA receptor antagonists)

Excitotoxicity refers to the process where excessive stimulation by neurotransmitters, such as glutamate, leads to nerve cell damage and death. This occurs when there is over-activation of receptors, particularly the ionotropic N-methyl-D-aspartate (NMDA) receptors. Such over-activation results in an influx of calcium ions into cells, causing oxidative stress and ultimately leading to cell death (110–112). Glutamate excitotoxicity via NMDA receptors plays a vital role in RGC death in glaucoma, often accompanied by oxidative stress and NLRP3 inflammasome activation. Moreover, inhibitors targeting the RIP1/RIP3/MLKL pathway, such as GSK872 and necrostatin-1, have shown significant neuroprotective effects by mitigating RGC necroptosis. These inhibitors not only prevent necroptotic cell death but also play a crucial role in suppressing NLRP3 inflammasome activation, thereby reducing neuroinflammation and neuronal damage in models of glutamate-induced excitotoxicity (113).

A promising area of research focuses on mitigating excitotoxic damage through NMDA receptor antagonists. Studies have demonstrated that compounds such as Philanthotoxin-343 can safeguard RGCs from NMDA-induced excitotoxicity, thereby preserving visual function in animal models (114). Beyond pharmacological interventions, growing evidence suggests that microRNAs play a crucial role in regulating excitotoxicity. For instance, miR-145-5p has been implicated in RGC apoptosis via the PI3K/AKT signaling pathway, highlighting the potential of modulating microRNA expression as a neuroprotective strategy (115). Furthermore, the use of microRNA mimics has shown promise in reducing RGC death following NMDA exposure, suggesting that genetic approaches may serve as a complementary avenue alongside pharmacological treatments (116).

Research indicates that glutamate levels in the vitreous humor of the eyes of humans, dogs, and rabbits with glaucoma are elevated, reaching concentrations that could be harmful to RGCs (110). Additionally, memantine, a substance known as a non-competitive NMDA receptor antagonist, may provide protective effects against glaucomatous visual neuropathy (117, 118).

Despite the promise of targeting excitotoxic pathways, clinical outcomes have been inconsistent, indicating that the success of these treatments may vary depending on the context of RGC degeneration (119). This suggests a need for further investigation into the specific mechanisms and factors that contribute to excitotoxic damage in glaucoma. One such factor is reduced retinal blood flow, which exacerbates ischemia, causing the accumulation of glutamate and other neurotoxic substances and creating a hypoxic environment in the retina (112). This hypoxia not only promotes oxidative stress but also impairs the availability of essential neurotrophic factors like brain-derived neurotrophic factor (BDNF), which are critical for RGC survival (112). In the absence of sufficient neurotrophic support, RGCs become more vulnerable to apoptotic signals triggered by excitotoxicity, amplifying neuronal damage and contributing to the progressive vision loss characteristic of glaucoma (111).

Moreover, neuroinflammation further complicates this process. Activated microglia and astrocytes release pro-inflammatory cytokines that not only exacerbate oxidative stress but also disrupt neurotrophic factor signaling, creating a detrimental feedback loop. This cycle reduces the availability of neurotrophic factors, making RGCs even more susceptible to excitotoxic damage (120, 121). Müller cells, in particular, play a pivotal role in this process by initiating microglial activation through the ATP/P2X7 receptor pathway, leading to increased production of pro-inflammatory mediator (122). This triggers a harmful feedback loop where activated microglia, in turn, stimulate Müller cells, further intensifying the inflammatory response (122, 123). As neuroinflammation progresses, glial cells release cytotoxic factors, disrupt the extracellular matrix, and contribute to glutamate receptor-mediated excitotoxicity, all of which accelerate RGC loss (123).

#### 2.2.2 Reduction of the mitochondrial dysfunction and oxidative stress impact

Mitochondrial dysfunction plays a crucial role in the pathophysiology of glaucoma by disrupting oxidative phosphorylation and increasing the production of reactive oxygen species (ROS). When the cellular antioxidant system fails to neutralize excess ROS, oxidative stress ensues, leading to RGCs degeneration and optic nerve damage (124). Elevated IOP has been shown to exacerbate oxidative stress by reducing the activity of key antioxidant enzymes, such as glutathione peroxidases, which are essential for combating oxidative damage (125, 126). Additionally, mitochondrial defects contribute to increased ROS levels and decreased ATP production, which is vital for maintaining cellular homeostasis, particularly in energy demanding cells like RGCs (1, 127). The relationship between oxidative stress and mitochondrial dynamics is also significant. Oxidative stress-induced mitochondrial fission has been implicated in RGC degeneration, with the loss of proteins such as AKAP1 leading to Drp1-mediated mitochondrial fission, increased ROS production, and diminished ATP synthesis, further contributing to RGC death (127, 128). Additionally, oxidative stress can trigger mitochondrial membrane potential collapse, leading to apoptosis and progressive neuronal loss in glaucoma (129). Given that oxidative stress is a significant factor in glaucoma, antioxidants may serve as a beneficial therapeutic approach, especially in the early stages of the disease, before irreversible damage occurs. Numerous studies have explored the effectiveness of antioxidants in treating glaucoma, using various compounds such as vitamins, coenzyme Q10, astaxanthin, Ginkgo biloba, omega-3/6 fatty acids, and resveratrol in animal models (124).

Research has shown a strong association between oxidative stress and the activation of apoptotic pathways in RGCs, suggesting that antioxidants may offer neuroprotective benefits. One example is thioredoxin 2 (Trx2), a crucial mitochondrial antioxidant, which has been shown to promote RGC survival by reducing oxidative stress and apoptosis in experimental glaucoma models (130). Alternatively, the application of AAV-NDI1 gene therapy has demonstrated remarkable efficacy in murine glaucoma models by directly enhancing mitochondrial function, significantly reducing ROS levels, and ultimately preserving RGCs from degeneration (131).

Vitamin E deficiency, for instance, has been shown to exacerbate lipid peroxidation and RGC loss in rat models of glaucoma, suggesting that sufficient vitamin E intake could benefit individuals at risk for or diagnosed with glaucoma. However, more research is necessary to confirm the efficacy of vitamin E supplementation as an adjunct treatment for glaucoma, particularly through human clinical trials (132). Similarly, resveratrol (RES), a polyphenol found in peanuts and red grapes, has demonstrated potential antioxidant and neuroprotective properties. Studies suggest that intraperitoneal injection of RES may inhibit the apoptotic Bax-caspase-3 pathway, thereby preventing RGC death. Additionally, RES appears to reduce reactive gliosis and may have neuroprotective effects by decreasing the production of pro-inflammatory chemicals in the retina following retinal ischemia/reperfusion (133).

Naringenin, a flavonoid found in citrus fruits like grapefruits, has also been studied for its potential to mitigate age-related retinal degeneration by influencing mitochondrial dynamics and autophagy (134). Beyond animal studies, several antioxidants are currently undergoing clinical trials to evaluate their effectiveness in human patients. For instance, Ginkgo biloba, a traditional Chinese medicinal plant, has been studied for its antioxidant and neuroprotective properties (135). A 2018 study reported that glaucoma patients who took dietary supplements containing vitamins, minerals, omega-3 fatty acids, and plant extracts including Ginkgo biloba experienced improved blood flow and reduced retinal artery pressure after 1 month of treatment (136).

Moreover, recent research highlights the intricate interplay between oxidative stress and other pathological mechanisms in glaucoma. Specifically, the combined effects of oxidative stress and endoplasmic reticulum stress have been linked to the worsening of glaucomatous damage to both RGCs and the optic nerve (137). This multifactorial nature of glaucoma suggests that a comprehensive approach targeting both mitochondrial dysfunction and oxidative stress, alongside traditional IOP-lowering therapies, may be necessary for effective treatment (138, 139).

Mitochondrial dysfunction is a major contributing factor in the pathophysiology of glaucoma. Coenzyme Q10 (CoQ10), an antioxidant found in mitochondria, plays a crucial role in the electron transport chain. Its neuroprotective effects have been demonstrated in conditions such as cerebral ischemia and neurodegenerative disorders (140). CoQ10 has been shown to reduce pathogenic glutamate levels following reperfusion in animal models of acute IOP increases, potentially contributing to neuroprotection (141). Two ongoing randomized clinical trials are comparing CoQ10 supplementation with placebo in individuals with primary open-angle glaucoma who are being treated with IOP-lowering drugs. One trial (Phase IV, NCT04038034) is testing oral CoQ10 supplementation to assess its effects on structural and functional changes. The second trial (NCT03611530) is comparing the time to disease progression between patients receiving placebo and those using eye drops containing both CoQ10 and vitamin E (19). Additionally, glutamate, another antioxidant, has been studied in patients with POAG and normal-tension glaucoma (NTG). Findings suggest that a decrease in glutamate levels in the AH is associated with visual field loss and may be involved in glaucoma pathophysiology. In NTG patients, a reduction in this antioxidant can lead to optic nerve damage and RGC loss even in the absence of elevated IOP (142).

#### 2.2.3 Modulation of the immune response

Proper management of neuroinflammation is crucial to safeguarding neurons from inflammatory damage and promoting natural healing processes. Given the prevalence of neuroinflammation in RGCs during glaucomatous neurodegeneration, immunomodulation presents a potentially effective treatment strategy. Glial cells, being key players in neuroinflammation, serve as promising targets for improving outcomes in glaucoma and restoring immunological homeostasis. In glaucomatous neurodegeneration, astroglial reactivity persists longer than the transient action of microglia, highlighting their importance as therapeutic targets (143). The success of interventions targeting microglia in the retina or optic nerve head to reduce neurodegeneration underscores the need for early intervention (144–146).

Microglial activation in glaucoma is closely linked to the release of pro-inflammatory cytokines and chemokines, which exacerbate RGC damage. Elevated IOP has been shown to upregulate interleukin-1 (IL-1), suggeting that astrocytes and Müller cells may initiate this inflammatory cascade, leading to microglial activation and subsequent neuronal injury (147, 148). The Janus kinase-signal transducer and activator of transcription (JAK-STAT) signaling pathway further mediates this inflammatory response in chronic glaucoma (149, 150). Astrocytes also play a crucial role in the neuroinflammatory environment by undergoing reactive gliosis, marked by increased glial fibrillary acidic protein (GFAP) expression, which can compromise the blood-retinal barrier and promote inflammation (149, 151). Their reactive state can be influenced by activated microglia, potentially shifting astrocytes from a neuroprotective to a neurotoxic phenotype (152). The crosstalk between microglia and astrocytes is therefore a key determinant of whether immune responses in glaucoma contribute to neuroprotection or neurodegeneration. Molecular mechanisms driving this immune activation include toll-like receptors (TLRs) and the complement system, both of which mediate microglial responses to stressors such as elevated IOP and systemic inflammation (153). Specifically, TLR4 signaling has been implicated in microglial activation and neuroinflammation in glaucomatous retinas, while the NLRP3 inflammasome plays a role in caspase-1-mediated pathways that can further exacerbate neuronal injury (154–156).

In addition to the exploration of these mechanisms, recent studies have highlighted the potential of specific microRNAs and the gut microbiota in modulating immune responses in glaucoma. miR-146a, for instance, has been shown to reduce neuroinflammation by promoting an anti-inflammatory phenotype in activated microglia, offering a novel therapeutic target to mitigate RGC damage (157). Furthermore, alterations in gut microbiota composition have been linked to immune dysregulation in glaucoma. The gut-ocular axis hypothesis suggests that dysbiosis may trigger systemic immune responses, exacerbating ocular inflammation and contributing to glaucoma progression (158). These emerging areas of research emphasize the need to consider systemic factors, such as microRNAs and gut health, in the development of comprehensive therapeutic strategies for glaucoma.

Research has identified several potential therapeutic targets for mitigating neurodegeneration by modulating inflammatory responses. For example, ibudilast, a cAMP phosphodiesterase inhibitor, has been shown to reduce pro-inflammatory mediators and enhance neuronal survival in hypertensive rat eyes, emphasizing the importance of targeting neuroinflammation (159). Additionally, the prevention of monocyte entry into the optic nerve head through irradiation has been demonstrated to protect against neuronal damage in DBA/2J mice, further illustrating the role of immune cells in neurodegenerative processes (160). Moreover, studies suggest a connection between neuroinflammation and pathological changes at the node of Ranvier in DBA/2J glaucoma. The protective effects of immunosuppressive treatment with fingolimod offer new therapeutic avenues (161). Impairments in central and effector CD4 memory cells in primary POAG patients, along with reduced T cell activation thresholds, indicate that T cells actively contribute to pro-inflammatory signaling (162). Developing immunological tolerance to glaucoma-associated antigens could be a viable strategy for controlling inflammatory responses and potentially slowing disease progression. Increasing the number of regulatory T cells (Tregs), which inhibit immune responses, has been shown to reduce vision loss in mice with glaucoma, suggesting that immunomodulatory drugs targeting T-cell activity could benefit some patients (163).

Tumor necrosis factor-alpha (TNF-α)-induced neurotoxicity plays a significant role in the neurodegenerative damage observed in glaucoma (164). Elevated levels of TNF-α in the AH of individuals with glaucoma, compared to controls, facilitate the apoptosis of RGCs via interaction with TNF-receptor-1 (TNF-R1). In rats, intravitreal injection of TNF-α correlates with optic nerve degeneration and delayed death of retinal neuronal cells, associated with increased expression of NF-κB p65 in the optic nerve, suggesting a link to TNF-α-induced axonal degeneration (165). Therefore, inflammatory suppressants or TNF-α antagonists like Etanercept should be considered as adjunct therapy for glaucoma (147). Protecting RGCs effectively requires targeted inhibition of cell death signaling and/or enhancement of survival signaling without compromising TNF-α-triggered survival pathways (164). TNF-α antagonists, such as infliximab, have emerged as promising options due to their ability to mitigate the neuroinflammatory processes that contribute to RGC apoptosis and optic nerve degeneration. To further explore the potential of this therapeutic approach, a clinical trial (NCT05180994) is currently being conducted involving patients undergoing their first penetrating keratoplasty (PKP) surgery. This trial aims to assess the safety, efficacy, and therapeutic potential of topical infliximab eye drops in preventing post-surgical glaucoma. The study will closely monitor and compare key outcomes, such as retinal nerve fiber thickness, glaucoma incidence, visual acuity, ocular surface inflammation, epithelial healing time, and overall ocular health between the infliximab-treated and control groups. Furthermore, a study by Yang et al. explored the role of oxidative stress in the neuroinflammation and neurodegeneration associated with glaucoma. The results indicated that antioxidant treatment reduced neuroinflammation in mice with OHT, while mice deficient in the antioxidant enzyme superoxide dismutase 1 (SOD1) showed increased inflammation compared to controls. These findings highlight the importance of oxidative stress in neuroinflammation and suggest that antioxidant therapy could be a potential strategy for neuroprotection in glaucoma (166). Additionally, there appears to be a potential link between glaucoma-related neurodegeneration and autoimmune disease. A promising therapeutic approach could involve focusing on immunological alterations, such as antibodies against γ-synuclein. Targeting these immune responses specifically in the retina of glaucoma patients might help protect RGCs and potentially slow disease progression (167).

#### 2.2.4 Altering the glial cell activity

Glial cells are categorized into two types: macroglial cells and microglial cells. Microglial cells function as immunocompetent cells and are involved in cell death and apoptosis. Macroglial cells produce cytokines, including TGF-α, ciliary neurotrophic factor (CNTF), and platelet-derived growth factor (PDGF) (168, 169). CNTF has been demonstrated to prevent cell degeneration following axotomy and promote neurite growth and neuronal survival (170). Given their involvement in these processes, glial cells are emerging as promising therapeutic targets due to their potential for neuroprotection and optic nerve regeneration (171).

Studies have shown that patients with primary open-angle glaucoma have lower CNTF concentrations in their tear fluid and AH, especially those experiencing significant vision loss (172). Moreover, the relationship between glial cell modulation and the molecular mechanisms underlying RGC death is multifaceted. Increased oxidative stress, deprivation of neurotrophic factors and reduced blood flow are all implicated in RGC degeneration in glaucoma. For example, neurotrophic factors such as nerve growth factor (NGF) are essential for RGC survival, and their deprivation can lead to increased apoptosis (173, 174). In addition, studies have shown that glial cells can influence vascular dynamics in the retina, which is essential for maintaining adequate blood flow and nutrient delivery to RGCs (171, 175). Impaired ocular blood flow, often linked to glial activation, can exacerbate the vulnerability of RGCs, indicating that restoring normal glial function may help mitigate these effects (176, 177). Furthermore, intravitreal PDGF infusion has been found to prevent retinal ganglion cell loss. This neuroprotective effect is mediated by astrocytes and amacrine cells, which release protective substances in response to PDGF exposure (178, 179). NT-501, a therapy involving encapsulated cell technology (ECT) intravitreal implants, delivers therapeutic agents directly into the vitreous humor for a duration of one to one-and-a-half years. These implants contain immortalized pigment epithelial cells transfected with the human CNTF gene. The efficacy and safety of NT-501 have been assessed in two 1-year Phase II randomized clinical trials in patients with early and advanced retinitis pigmentosa (RP), as well as a 6-month Phase I non-randomized clinical trial in RP patients. The trials reported positive outcomes in terms of tolerability and visual function (180–183). Additionally, a 1-year randomized Phase II clinical trial in patients with atrophic macular degeneration showed a favorable safety profile and benefits for vision maintenance (184). Current Phase I and II randomized clinical trials are ongoing to evaluate the safety and efficacy of NT-501 in patients with POAG (185).

#### 2.2.5 Nicotinamide

Nicotinamide, also known as vitamin B3, has been extensively studied for its potential to serve as a neuroprotective agent in the treatment ofglaucoma. Extensive research across various animal models has demonstrated its ability to mitigate optic nerve damage, axon loss, and retinal vascular damage, suggesting its potential to RGCs and their axons (186, 187). One of the key mechanisms through which nicotinamide may exert these protective effects is by restoring the levels of nicotinamide adenine dinucleotide (NAD+), which is critical for mitochondrial metabolism and energy production. NAD+ depletion is associated with RGC apoptosis and neurodegeneration, both of which are central to the progression of glaucoma. Supplementing with nicotinamide has been shown to significantly increase NAD+ levels, thereby protecting RGCs from metabolic dysfunction and preventing cell death (188–190). This restoration of NAD+ helps preserve RGC function and structure in animal models of glaucoma, especially in the face of elevated IOP and metabolic stress (187, 191, 192). Another important aspect of the neuroprotective properties of nicotinamide is its antioxidant effect. Glaucoma is associated with increased oxidative stress, where ROS accumulate and cause cellular damage and apoptosis. Nicotinamide acts as an antioxidant by neutralizing these ROS, thereby shielding RGCs from oxidative damage (138). Additionally, it has been found that nicotinamide can influence the expression of antioxidant enzymes, further boosting retinal resistance to oxidative stress (190). Furthermore, nicotinamide may have indirect anti-inflammatory effects, which are particularly relevant in glaucoma. Nicotinamide appears to modulate the activity of glial cells like microglia and astrocytes, reducing the release of pro-inflammatory cytokines and mitigating their damaging effects on RGCs (193, 194). Studies have also shown that nicotinamide supplementation can enhance RGC function, as evidenced by improved PERG responses, which can detect early functional impairments before structural changes occur (195, 196). Recent findings have further supported these benefits, showing that oral nicotinamide administration provides robust, dose-dependent neuroprotection of RGCs in rat glaucoma models. Higher doses were particularly effective in preserving RGC structure and metabolism, reinforcing the potential of nicotinamide as a therapeutic agent for glaucoma-induced neurodegeneration (197). This indicates that nicotinamide not only helps preserve RGC viability but also supports overall retinal function in glaucoma.

Eight clinical studies of nicotinamide supplementation in glaucoma are exploring its potential neuroprotective and therapeutic effects, although results vary due to different stages of advancement. Several studies, including (NCT06731582), (NCT05275738), and (NCT06078605), are still under recruitment or in progress, so their main results are not yet available. However, (NCT05916066), a completed study, has shown that glaucoma patients have lower plasma nicotinamide levels than healthy controls, and that short-term nicotinamide treatment appears to affect retinal vascularization, suggesting possible retinal health benefits. The current study (NCT05695027) is investigating the combination of nicotinamide and pyruvate for open-angle glaucoma, while the Phase 3 study (NCT05405868) aims to assess the efficacy of nicotinamide in slowing visual field loss in glaucoma patients. In addition, a study (NCT06333236) is exploring the effects of nicotinamide-based treatments in combination with other compounds but results are not yet available. Overall, while initial results are promising, particularly with regard to retinal health and visual function, further research is needed to fully establish the benefits and safety of nicotinamide supplementation for glaucoma management.

#### 2.2.6 Statins

Statins have been demonstrated to enhance blood circulation and modulate immune responses within the nervous system (198, 199). Research involving animal models has shown that statins can extend the lifespan of RGCs in response to OHT by reducing apoptosis (200, 201). The exact mechanisms behind the potential protection of statins against glaucoma are still being studied. Statins may influence the Rho kinase pathway (202) and affect the TM, which regulates AH outflow (203), but further research is needed to fully understand these effects. *In vitro* studies have investigated the neuroprotective effects of statins, particularly focusing on RGCs and their progressive loss due to apoptosis in glaucoma. These studies revealed that statins inhibit gliosis and RGC apoptosis in Müller cells, with TNF-α identified as a critical mediator. These findings suggest that statins could represent a promising therapeutic option for glaucoma treatment (204). Studies like those by Song et al. support this, demonstrating atorvastatin's effectiveness in reducing IOP in ocular hypertension models (205). Additionally, elevated lipid levels in patients with normal tension glaucoma suggest that statins may offer protective effects by improving lipid profiles and modulating oxidative stress, potentially influencing the pathogenesis of these glaucoma subtype by restoring the balance of high-density lipoprotein (HDL)-linked paraoxonase 1 (PON1) enzymatic activity and mitigating dyslipidemia (206).

Recent studies have investigated the relationship between statin use and glaucoma, producing varying outcomes. A recent study analyzed data from the All of Us Research Program, encompassing 79,742 adults with hyperlipidemia (207). The findings revealed that statin use was linked to a higher likelihood of developing glaucoma. This association was particularly pronounced among individuals aged 60 to 69 years, as well as those with optimal or elevated LDL-C levels (207). These results suggest a potential risk factor, highlighting the need for further investigation into the effects of statins on glaucoma.

Additionally, statins may provide protective benefits against open-angle glaucoma. A 2012 study analyzed the relationship between statin use and OAG among nearly 500,000 patients with hyperlipidemia enrolled in managed care programs. The study found that patients who adhered to a statin regimen for 2 years were 8% less likely to develop open-angle glaucoma compared to those who did not use statins (208). Another clinical study (NCT00913562) aimed to assess whether rosuvastatin could improve endothelial function in these patients, using flow-mediated vasodilatation of the brachial artery and flicker-induced vasodilatation of retinal vessels. However the study has been withdrawn.

#### 2.2.7 Epigallocatechin-3-gallate

Epigallocatechin-3-gallate (EGCG), a potent flavonoid found in green tea, has received significant attention for its neuroprotective properties in glaucoma. Its mechanisms of action include anti-inflammatory, antioxidant, and anti-apoptotic effects, which collectively contribute to RGC preservation and disease mitigation (209). One of EGCG primary neuroprotective mechanisms is its ability to suppress neuroinflammation, a key contributor to glaucoma progression. It inhibits the expression of pro-inflammatory cytokines such as IL-4, IL-6, TNF-α, and IL-1β, while also downregulating the nuclear factor-κB (NF-κB) signaling pathway, a central regulator of inflammatory responses (210–212). In a rat model of optic nerve injury, EGCG administration reduced inflammation and optic nerve damage, further reinforcing its therapeutic potential (210). In addition to its anti-inflammatory effects, EGCG is a powerful antioxidant that eliminates free radicals and protects RGCs from oxidative stress, a major contributor to neurodegeneration in glaucoma. Studies have demonstrated that EGCG reduces oxidative damage in neuronal cells and improves RGC survival in animal models with elevated IOP (209, 213, 214). Furthermore, its ability to cross the blood-brain barrier enhances its efficacy as a neuroprotective agent (215). Beyond its anti-inflammatory and antioxidant properties, EGCG modulates key signaling pathways that regulate neuronal survival. It activates the hedgehog signaling pathway, which has been associated with neuroprotection against corticosterone-induced neurotoxicity (216). Additionally, it influences the phosphatidylinositol-3-kinase (PI3K)/protein kinase B (AKT) and mitogen-activated protein kinase (MAPK) pathways, both of which play critical roles in cell survival and apoptosis regulation (217). In an optic nerve crush model, EGCG treatment significantly increased RGC survival and maintained higher neurofilament triplet L protein expression (218).

Building on preclinical evidence, a clinical study (NCT00476138) was conducted to evaluate EGCG impact on retinal function in patients with ocular hypertension or early glaucoma. This randomized, placebo-controlled trial included 40 participants divided into two groups: a treatment group receiving 200 mg/day of oral EGCG alongside standard beta-blocker therapy, and a placebo group receiving only beta-blockers. Patients underwent comprehensive ophthalmic evaluations at baseline, 3 months, and 6 months, including Humphrey perimetry, PERG recordings, and RNFL thickness measurement using OCT. The main outcome measure was PERG amplitude, a key indicator of RGC function. Secondary measures included perimetric indices and RNFL thickness. In a cross-over design, patients switched between placebo and EGCG treatment after 6 months, allowing further assessment of EGCG's effects. PERG recordings were obtained using high-resolution electroretinography, capturing retinal responses to counterphased horizontal gratings. OCT imaging provided detailed RNFL thickness measurements, with multiple scans ensuring accuracy and reproducibility. While the results of this clinical study have been submitted for publication, they are not yet publicly available. However, preliminary findings suggest that EGCG supplementation may enhance RGC function and help preserve RNFL thickness, further supporting its potential role in glaucoma management.

#### 2.2.8 Forskolin

Forskolin, a labdane diterpenoid derived from *Plectranthus barbatus* (formerly *Coleus forskohlii*), is a direct activator of adenylyl cyclase, increasing intracellular cyclic AMP (cAMP) which play a crucial role in IOP regulation, neuroprotection, and cellular signaling (219–221). Forskolin effectively lowers IOP by decreasing AH production, a key therapeutic target in glaucoma (222). Clinical trials have demonstrated that 1% forskolin eye drops significantly reduce IOP in patients with open-angle glaucoma, offering a potential alternative or adjunct to traditional treatments (223). A 2012 randomized controlled trial also examined the effects of an oral supplement containing forskolin and rutin in patients with POAG, who were already receiving maximum tolerated medical therapy. The study found that the supplement helped improve IOP control and led to a modest reduction in IOP (224). Beyond its IOP-lowering effects, forskolin exhibits direct neuroprotective properties. It enhances RGC survival by mimicking neurotrophin activity and modulating critical pathways such as PI3K/Akt and GSK-3β inhibition, reducing calpain activity, and mitigating glutamate-induced neurotoxicity (225–227). Studies indicate that combining forskolin with other neuroprotective agents enhances its efficacy. In rodent models of hypertensive glaucoma, dietary supplementation with forskolin, homotaurine, and B vitamins significantly protected against RGC degeneration (228). Similarly, forskolin combined with L-carnosine exhibited synergistic neuroprotection in a rat model of retinal ischemia (227).

A clinical study (NCT01254006) investigating RNFL modifications in glaucoma patients is currently in the recruiting phase. Meanwhile, two other clinical trials, one examining the effects of forskolin on IOP in glaucomatous patients (NCT00864578) and another evaluating its effects in patients undergoing treatment with beta-blockers or prostaglandin eye drops (NCT00863811), have been withdrawn.

### 2.3 Long-acting and personalized therapies

#### 2.3.1 Prolonged medication delivery mechanisms, or extended-release formulations

The management of ocular surface disease and patient adherence in glaucoma treatment have significantly improved with the introduction of innovative drug delivery technologies. Traditional therapies, primarily topical eye drops, are often associated with low adherence due to complex dosing regimens, side effects, and challenges with self-administration (229, 230). To address these limitations, advanced drug delivery systems have been developed to enhance therapeutic efficacy while reducing patient burden.

A significant limitation of conventional eye drops is their low ocular bioavailability, meaning that only a small fraction of the administered drug reaches the target site within the eye, thereby diminishing therapeutic efficacy. Advances in drug delivery systems, such as gel formulations, *in situ*-forming gels, and nanoparticles, present promising solutions to this issue. *In situ* gels transition from a liquid to a gel state upon contact with the ocular surface, enhancing drug bioavailability by prolonging the drug's residence time. These gels can be triggered by changes in temperature, pH, or ion exchange, and offer benefits like improved drug retention, extended release, and better patient compliance (231, 232).

Nanoparticles, which include lipid-based nanoparticles, polymeric nanoparticles, and dendrimers, encapsulate drugs to protect them from degradation and enable controlled release. Examples of these include chitosan nanoparticles, PLGA [poly(lactic-co-glycolic acid)] nanoparticles, and lipid-based carriers such as liposomes and solid lipid nanoparticles (231, 233). Additionally, some advanced drug delivery systems combine the features of in situ-forming gels and nanoparticles to maximize the advantages of both, resulting in enhanced drug delivery performance and bioavailability, as seen in nanoparticle-loaded gels (231).

The use of biodegradable implants, such as Bimatoprost SR and titanium intraocular implants loaded with travoprost, represents one of the most promising strategies for managing persistent glaucoma. These devices provide sustained reductions in IOP, potentially delaying the need for more invasive surgical interventions (234, 235). Clinical trials have shown that these implants allow for continuous drug delivery, reducing the reliance on daily eye drops and improving patient adherence to treatment (235, 236). For example, the travoprost intracameral implant has significantly decreased the need for topical medications, thereby enhancing patient compliance (230, 236).

Currently, bimatoprost SR is undergoing Phase III trials across six studies. Of these, one study has been completed (NCT02247804), which compared the safety and efficacy of bimatoprost SR with timolol administered topically twice daily. Two other studies are comparing bimatoprost SR with selective laser trabeculoplasty (NCT02636946 and NCT02507687), while the remaining three are focused on assessing the long-term efficacy and safety of bimatoprost SR (NCT03850782, NCT03891446, and NCT02250651).

Similarly, three ongoing trials are evaluating a travoprost-filled titanium intraocular implant in comparison with topical timolol therapy (NCT02754596, NCT03868124, and NCT03519386). A biodegradable intracameral implant for extended-release travoprost has been tested for up to 24 months, but it has been associated with side effects such as ocular hyperemia, photophobia, anterior chamber inflammation, cataract formation, and corneal endothelial cell loss (NCT02371746).

A promising alternative is the development of contact lenses as medication delivery devices. These lenses offer the potential to enhance bioavailability and reduce the frequency of administration, enabling prolonged release of pharmaceuticals (237, 238). Research indicates that, compared to conventional eye drops, contact lenses significantly improve medication retention on the ocular surface, leading to enhanced therapeutic effects (239, 240). Furthermore, advances in materials science have made it feasible to incorporate micelles and nanoparticles into contact lenses, which not only boosts patient adherence but also allows for controlled and sustained medication release (241, 242).

Intracanalicular devices, such as OTX-TP and Evolute hydrogel punctum plugs, represent another innovative approach to drug delivery. These devices allow for localized drug release, minimizing systemic exposure and side effects while maximizing therapeutic efficacy (243). Studies have shown that these systems effectively maintain therapeutic drug levels in the eye, enhancing treatment adherence and reducing the incidence of ocular surface disease associated with conventional therapies (243, 244). The gel-forming drops include SoliDrop and DuraSite ISV-215, which contain Brimonidine and Bimatoprost, utilizing excipients like poly(lactic-co-glycolic acid) and Pentablock copolymer; both are in preclinical stages (245, 246). An ocular insert specifically for Bimatoprost is being tested in a Phase II clinical trial, utilizing a silicone matrix and polypropylene in the conjunctival cul-de-sac (247). These formulations help extend the contact time with the ocular surface, thereby boosting the therapeutic efficacy of glaucoma treatments (229, 242). For instance, products like SoliDrop and DuraSite ISV-215 have demonstrated improved patient compliance by decreasing the frequency of administration needed for effective IOP management (229, 242).

Intracameral implants, including DURYSTA, iDose Travoprost, ENV515 or Travoprost XR OTX-TIC, PA5108, and DE-117, involve multiple formulations with various polymers, progressing through Phase I to Phase III clinical trials (248–254). Lastly, subconjunctival injections, such as Durasert™ and POLAT-001, are also in the clinical trial stages for sustained release of latanoprost (255–257).

#### 2.3.2 Stem cell-based therapies

Stem cell therapy is emerging as a groundbreaking approach for treating glaucoma, focusing on directly repairing the TM, which is responsible for regulating IOP. Unlike traditional treatments that manage symptoms, stem cell therapy aims to restore TM function, offering long-term benefits.

There are two key types of stem cells driving this advancement: human pluripotent stem cells (hPSCs) and mesenchymal stem cells (MSCs). hPSCs have the remarkable ability to differentiate into any cell type, enabling the repair or regeneration of damaged tissues. According to the International Society for Cell and Gene Therapy (ISCGT), MSCs are multipotent stem cells capable of generating new cells and differentiating into other cell types (258, 259). Research has also advanced the reprogramming of adult cells to return to a pluripotent state through molecular manipulation, producing induced pluripotent stem cells (iPSCs). The production of iPSCs typically involves using viruses to deliver genes encoding specific transcription factors into adult cells (260).

Research has demonstrated the ability of TM stem cells (TMSCs) to migrate toward the TM and develop into TM cells when injected into the anterior chamber of a healthy mouse eye. This process helps restore TM function, crucial for regulating IOP (261).

An *in vivo* study in mice and rats has shown that MSCs and their secreted factors play a crucial role in tissue repair in OAG by reactivating local neuronal progenitors. Additionally, laser treatment has emerged as a promising method to facilitate progenitor cell recruitment by MSCs, thereby enhancing tissue repair in chronic diseases (262). Adult stem cells found in a specific region of the eye known as the Schwalbe's line exhibit unique properties, making them promising candidates for developing glaucoma treatments. Research by Braunger et al. involved treating four cynomolgus monkeys with the nucleotide analog bromodeoxyuridine (BrdU) for 4 weeks, identifying cells with long-term retention of BrdU and OCT4 immunoreactivity. These cells, part of a niche in the Schwalbe's line region, represent endogenous adult stem cells that can differentiate into functional TM cells, potentially regenerating or repairing damaged TM, improving AH outflow, and reducing IOP (263).

Further studies have highlighted the neuroprotective effects of MSCs in glaucoma. MSCs, when injected into the anterior chamber of the eye, protect the optic nerve similarly to TMSCS (264, 265). To enhance the targeting of stem cells to the TM, scientists have employed magnetic nanoparticles to label MSCs, allowing them to magnetically guide to the TM after injection into mouse models. This method has been shown to be highly effective, preserving stem cell viability and their ability to differentiate into functional TM cells (266). An example of this technique is the use of adipose-derived mesenchymal stem cells (adMSCs) for restoring the TM (266, 267). Studies have shown that human adMSCs can differentiate into TM-like cells that not only resemble the morphology but also retain functional capabilities to regulate AH dynamics in mouse model *in vivo* (265). Since adMSCs are derived from the patient's own tissue and can be harvested in significant quantities through minimally invasive procedures, they are promising candidates for future clinical trials aimed at restoring TM function in glaucoma patients (268). Using reprogrammed stem cells derived from glaucoma patients (iPSCs), scientists can create cells resembling the natural ocular fluid drainage system, known as TM cells. When transplanted into the eye, these drainage-like cells could restore normal regulation of eye pressure. This personalized approach, using the patient's own cells, reduces the risk of rejection and provides a tailored treatment, potentially revolutionizing glaucoma therapy (269).

Studies have explored using MSC-derived exosomes to treat RGCs. These exosomes have been shown to deliver trophic and immunomodulatory substances, inhibit inflammatory cell migration, reduce the release of pro-inflammatory cytokines, and increase RGC survival (270, 271). Although bone marrow-derived MSC therapy shows promise as a treatment for retinopathies and glaucoma, safety studies involving human subjects are limited. A study investigating the safety and efficacy of using bone marrow stem cells to treat severe blindness caused by glaucoma involved two legally blind patients who received MSCs derived from their own bone marrow. The study aimed to assess potential side effects and measure improvements in vision, including visual field changes, sharpness, thickness of specific eye layers, and nerve cell function in the more severely affected eye. While the baseline electroretinography (ERG) findings showed typical characteristics of advanced glaucoma, there were no notable changes in ERG responses for either patient up to 1 week post-treatment. However, on day 15, one patient experienced a serious complication—retinal detachment with proliferative vitreoretinopathy—necessitating their removal from the trial. This complication is a known risk associated with certain ocular surgeries or procedures. The second patient maintained stable ERG responses throughout the study, indicating that the treatment did not worsen their condition, though it did not lead to significant improvement either (272).

Several clinical trials, including the Bone Marrow Derived Stem Cell Ophthalmology Treatment Study (SCOTS II), have explored the use of autologous bone marrow-derived stem cells (BMSC) for treating retinal and optic nerve conditions that cause irreversible vision loss. SCOTS II specifically focuses on conditions like macular degeneration, retinitis pigmentosa, and optic nerve damage from glaucoma or ischemic optic neuropathy. The trial administers stem cells through various injection routes, including retrobulbar, subtenon, intravitreal, intraocular, subretinal, and intravenous (NCT01920867 and NCT03011541). Another study, currently in the recruiting phase, will focus on evaluating the safety and potential therapeutic efficacy of intravenous and sub-tenon delivery of cultured allogeneic adult umbilical cord-derived mesenchymal stem cells (UC-MSCs) for treating eye diseases, with the goal of assessing improvements in patient outcomes and identifying any adverse effects over a 48-month period (NCT05147701). In addition, a study that is still in the planning phase will focus on evaluating the effectiveness of Platelet-rich Fibrin (PRF) membrane treatment in promoting wound healing in patients undergoing trabeculectomy for glaucoma (NCT06200727). Stem cells therapies for glaucoma are currently being tested, with the focus on the efficacy and safety of this strategy. There are also challenges to overcome, such as ensuring that transplanted stem cells survive, integrate properly and function. In addition, preventing immunological rejection and reducing the risk of tumor development are crucial issues. Despite these difficulties, stem cell therapy holds great promise for the treatment of glaucoma in the future, as it can halt the progression of the disease and improve the eyesight of sufferers.

#### 2.3.3 Gene therapy

Gene therapy presents significant potential for treating various eye diseases, including glaucoma, by targeting specific genetic factors involved in disease progression. In glaucoma, which is characterized by complex genetic backgrounds, gene therapy focuses on two primary aspects: neuroprotection of RGCs and reduction of IOP (273, 274). Viral vectors, particularly adenoviruses (AVs), adeno-associated viruses (AAVs), and lentiviruses (LVs), are commonly used in gene therapy studies for glaucoma due to their efficiency in delivering therapeutic genes to ocular cells (275). Although LVs are highly immunogenic, they offer a high capacity for transgenic expression and transfection efficiency. AAVs, notably AAV2, are less immunogenic, non-pathogenic, and particularly effective in transfecting RGCs (276–278).

Gene therapy strategies for glaucoma include both augmentation and silencing approaches. Augmentation involves introducing functional or modified genes to enhance cellular function, while inhibition aims to down-regulate genes associated with disease symptoms. Silencing strategies, such as siRNA and short hairpin RNA (shRNA), allow for precise gene regulation (279, 280). In the pursuit of IOP-targeted gene therapy, various approaches have been investigated, such as targeting the aquaporin 1 gene (281), the prostaglandin F2α receptor (282), RhoA kinase (283), exoenzyme C3 transferase (284, 285), metalloproteinase 1 (286), and myocilin (287). These approaches aim at modulating AH dynamics, TM function and ECM deposition to achieve sustained IOP reduction. In the most recent study, IOP was lowered in normotensive Brown Norway rats by delivering a transgene using a recombinant adeno-associated virus (rAAV) vector. The vector transgene appears to contain several elements, such as TC40, COX2, PTGFR, and TC45 that are linked to IOP modulation. Over the period of 1 year, the observed reduction in IOP ranged from 12.6% to 43.2%. The degree of reduction was dose-dependent, indicating that higher vector doses led to greater IOP reductions (282).

Gene therapy also offers a promising approach to targeting the TM, with the aim of improving AH outflow and reducing IOP (288). One potential approach involves the manipulation of long non-coding RNAs (lncRNAs) to modulate gene expression in TM cells under oxidative stress, potentially leading to improved IOP management (289). Elevated IOP in POAG is often linked to increased resistance to AH outflow due to excessive ECM accumulation and altered TM cell contractility (290, 291). Gene therapies targeting ECM components or the signaling pathways involved in TM cell contractility could restore normal outflow dynamics. For example, Rho-kinase inhibitors, which reduce TM cell contractility, have been shown to enhance AH outflow, suggesting that similar gene-based approaches could be effective in improving TM function and managing IOP (292).

Many neuroprotective gene therapy strategies for glaucoma focus on pathophysiological causes other than elevated IOP. Mitochondrial dysfunction plays a key role in glaucoma, leading to RGC death by disrupting energy metabolism and increasing the production of ROS. Recent studies have demonstrated the potential of gene therapy to improve mitochondrial function and mitigate oxidative stress. For example, eNdi1 gene therapy has been shown to significantly improve mitochondrial function in mouse models of glaucoma, effectively preserving RGCs by reducing levels of ROS (131). Similarly, neuroserpin gene therapy has demonstrated a protective role against oxidative stress by inhibiting RGC apoptosis and promoting functional preservation in glaucoma models (293).

In addition to mitochondrial pathways, gene therapy is also focusing on neuroprotection and the preservation of RGCs by preventing their degeneration. Neurotrophic factor deprivation, caused by a lack of neurotrophic factors, leads to reduced expression of genes linked to cell metabolism and axonal transport in animal models of glaucoma (294). A promising therapeutic target is BDNF, which is essential for neuronal survival, plasticity, and development, particularly in RGCs. In glaucoma, RGCs exhibit lower levels of BDNF and higher expression of its receptor, TrkB, which provides neuroprotection for RGCs. Direct administration of BDNF protein has been shown to reduce RGC apoptosis in animal models of glaucoma, paving the way for gene therapy studies using BDNF transgenes. Wójcik-Gryciuk et al. used AAV2-BDNF intravitreal augmentation gene therapy in a rat model of glaucoma and found that BDNF overexpression restored retinal TrkB levels, which were elevated due to RGC hypersensitivity to low BDNF levels (295). When different AAV2-BDNF constructs were used in gene therapy, Osborne et al. observed a decrease in cell apoptosis *in vitro*, comparable to that achieved with AAV2-BDNF alone or AAV2-TrkB. High-pressure glaucoma models in rats and optic nerve crush in mice employed similar approaches (296). Nishijima et al. focused on the intracellular domain of TrkB (iTrkB) for intravitreal gene therapy. Because iTrkB is smaller than other TrkB variants, it can be more effectively packaged into AAV vectors. This AAV design showed excellent results in various animal models of optic nerve injury, including glutamate/aspartate transporter knockout mice, silicone oil-induced glaucoma, and acute optic nerve compression (297). In an NMDA-induced excitotoxic mouse model, Shiozawa et al. used a tyrosine triple mutant AAV2 vector for BDNF gene delivery (tm-scAAV2-BDNF). This gene therapy improved ERG b-wave amplitude, decreased reactive gliosis in the retina, attenuated inner retinal thinning, and reduced RGC loss (298). In addition to BDNF, other neurotrophins and growth factors that have shown promise in experimental glaucoma treatments include vascular endothelial growth factor (299) and CNTF (300). Additionally, modulation of apoptosis and neurodegeneration pathways has been explored as a therapeutic strategy. Gene therapies targeting anti-apoptotic factors like Bcl-xL (301) and X-linked inhibitor of apoptosis protein (*Xiap*) (302) have shown neuroprotective effects in animal models of glaucoma. Similarly, interventions targeting Fas ligand (303) and nicotinamide mononucleotide adenylyltransferase 2 (Nmnat2) (304) have demonstrated RGC neuroprotection and preservation of visual function. Furthermore, Erythropoietin (EPO) gene therapy has shown promise in reducing inflammation and preserving RGC function (305). Additionally, targeting N-methyl-D-aspartate (NMDA) excitotoxicity (306) and regulating transcription factors like Max (307) and nuclear factor-E2-related factor 2 (Nrf2) (308) have demonstrated neuroprotective effects in preclinical studies. Finally, silencing gene therapies aiming at reducing IOP or promoting RGC neuroprotection have also been explored. These include targeting proteins involved in inflammatory pathways, such as IκB kinase (309) and Shp2 (310), as well as long non-coding RNAs like growth arrest-specific transcript 5 (GAS5) (311) and membrane proteins like Nogo-A and its associated receptor NogoR-1 (312).

A clinical study (NCT06465537) is ongoing to assess the safety, tolerability and preliminary efficacy of BD113vLVP in reducing IOP in patients with POAG and MYOC gene mutations. The study will also explore the characteristics of BD113vLVP metabolism and its potential impact on visual acuity. Despite the promising potential of gene therapy for the treatment of glaucoma, research bridging the gap between basic studies and clinical trials are still scarce, making further research in this field of ophthalmology crucial.

#### 2.3.4 Personalized medicine

Personalized medicine aims to tailor the prevention and treatment of glaucoma based on a patient unique genetic profile and other individualized characteristics. Research has shown that most cases of open-angle glaucoma are complex and polygenic, arising from the combined effects of multiple common genetic variations, with each contributing minimally to the overall disease (19). For instance, genome-wide association studies (GWAS) have identified numerous genetic loci that are associated with glaucoma risk factors, such as IOP, vertical cup-to-disc ratio, and central corneal thicknessrove the identification of individuals at high risk for vision impairment, it is critical to integrate these genetic findings with other known risk factors.

Machine learning algorithms have the potential to analyze large datasets, identifying the critical pathways that are disrupted in glaucoma and categorizing patients according to genetic similarities. This approach may reveal new biomarkers that can be used to diagnose, prognosticate, or develop targeted treatments for glaucoma. However, these biomarkers will need to undergo rigorous validation before they can be reliably used in clinical practice. Once validated, they could allow ophthalmologists to identify high-risk patients, enabling them to administer more aggressive treatments when necessary while avoiding unnecessary interventions for others (19).

Quantdicators like IOP and OCT measurements of retinal nerve fiber layer thickness are examples of biomarkers that are already being explored for their potential to track glaucoma progression. Moreover, advancements in imaging technologies, as well as genomic, metabolomic, and proteomic studies, are uncovering additional potential biomarkers. These will need to be validated across diverse patient populations, different stages of the disease, and ethnicities. Such biomarkers could eventually predict a patient's response to treatment, guide prognoses, or even serve as diagnostic tools. Recent studies have suggested, for example, that aqueous veins related to Schlemm's canal may serve as a structural biomarker to predict the outcomes of glaucoma surgery (19).

The personalized approach to glaucoma treatment emphasizes the integration of genetic profiles, environmental factors, and individual responses to therapy. By considering these elements, clinicians can tailor interventions to improve both treatment outcomes and patient adherence (313, 314). Recent studies have identified genetic factors that contribute to the development of glaucoma, such as variants in Caveolin 1 and 2, TMCO1, and CDKN2B-AS1 (315, 316). Additionally, polygenic risk scores (PRS) are emerging as an important tool to assess genetic predisposition, allowing for the stratification of individuals based on their risk level. This approach supports early intervention, particularly for those with ocular hypertension (317).

MicroRNAs (miRNAs), small non-coding RNA molecules, are also being explored as potential biomarkers for glaucoma. They have been found to be differentially expressed in the AH of glaucoma patients, which could offer insights for early diagnosis and disease monitoring (318). Furthermore, elevated levels of inflammatory and oxidative stress markers, such as TGF-β and VEGF, suggesting that inflammation plays a critical role in disease progression (319, 320). As research progresses and our knowledge of these factors increases, the possibility of developing more effective and personalized treatment options for glaucoma patients becomes increasingly feasible.

## 3 Conclusion and perspectives

**Figure 4:**
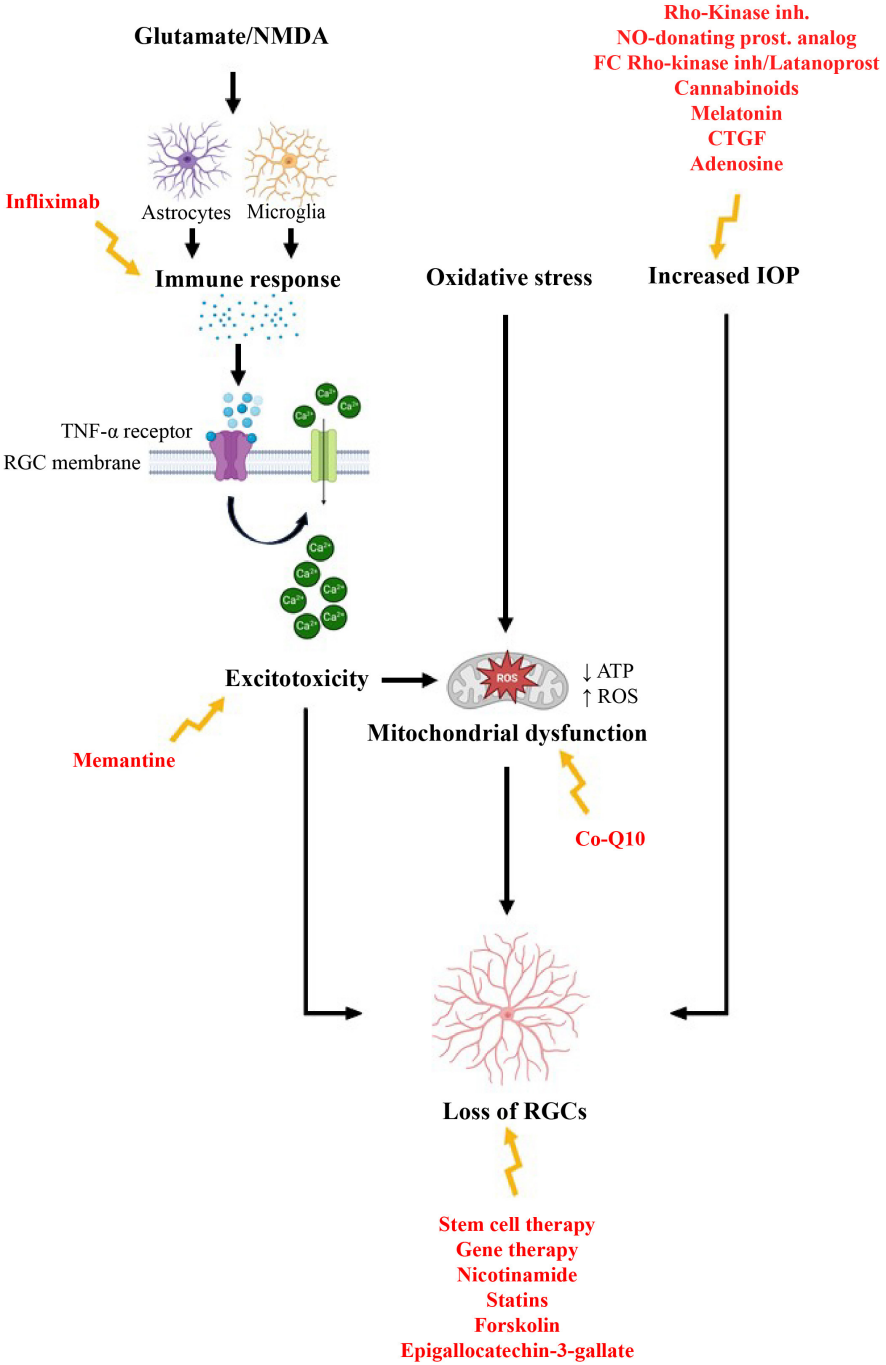
Developing therapeutic options addressing the underlying mechanisms of glaucoma [adapted and modified from Lambuk et al. (322) and Almasieh et al. (323)]. Figure designed partly with Biorender, https://app.biorender.com/illustrations.

Emerging glaucoma therapies are shifting toward a combination of IOP lowering treatments and neuroprotective strategies, signaling that effective management goes beyond reducing IOP alone. While traditional therapies have focused on lowering IOP to protect the optic nerve, this dual approach also aims to slow or prevent neurodegenerative processes, offering a more holistic solution to preserve vision. We have summarized the various strategies exposed in this review in the Table 1. By addressing both IOP reduction and neuroprotection, these therapies have the potential to improve long-term outcomes, offering not only symptomatic relief but also enhanced protection of the underlying neural structures. This evolution in glaucoma treatment could lead to a paradigm shift, increasing therapeutic efficacy, slowing disease progression, and more effectively preserving vision (Figures 3, 4).

**Table 1:** Overview of the therapeutical strategies presented in the present review.

**Expected effect**	**Therapeutic strategy**	**Therapy used**
Lower IOP	Increasing the AH outflow	Rho-kinase inhibitors; NO-donating prostaglandin analog; FC Rho-kinase inhibitor/latanoprost; Cannabinoids; Melatonin; Connective tissue growth factor; Adenosine; Gene therapy (aquaporin 1, RhoA kinase, myocilin)
	Decreasing the AH production	FC Rho-kinase inhibitor/latanoprost; Cannabinoids; Melatonin; Adenosine
	Decreasing the actomyosin contraction	Rho-kinase inhibitors
	Smooth Muscle Relaxation	NO-donating prostaglandin analog
	TGF-β regulation then decreasing fibrosis and TM function	Connective tissue growth factor, Gene therapy (aquaporin 1, RhoA kinase, myocilin)
	ECM remodeling	Connective tissue growth factor, Gene therapy (aquaporin 1, RhoA kinase, myocilin)
	Decreasing Cl- Channel activation	Adenosine
	Long-term TM regeneration	Stem cell therapy (MSCs); Stem cell therapy (hPSCs, iPSCs)
Neuroprotection/neuroregeneration	Reducing apoptosis and necroptosis	NMDA receptor antagonists; antioxidant; TNF-α antagonists; JAK-STAT inhibitors; miR-146a; statins; Forskolin, Stem cell therapy (MSCs), Exosome therapy (MSCs), Gene therapy [TrkB Activation, Silencing Long Non-Coding RNAs (GAS5), Bcl-xL and Xiap]
	Supporting neurotrophic survival mechanisms	NMDA receptor antagonists, gene therapy (BDNF overexpression, VEGF, CNTF)
	Reducing inflammation	NMDA receptor antagonists; antioxidant; TNF-α antagonists; JAK-STAT inhibitors; miR-146a; Gut microbiota modulators; Glial cell inhibitors; Nicotinamide; statins; EGCG; Exosome Therapy (MSCs); gene therapy [EPO, Silencing Long Non-Coding RNAs (GAS5)]
	Blocking excitotoxicity	NMDA receptor antagonists; Forskolin
	Reducing ROS	Antioxidant; TNF-α antagonists; Nicotinamide supplementation; statins; EGCG; Gene therapy (eNdi1)
	Enhancing retinal blood flow	Antioxidant; statins
	RGC metabolism	Nicotinamide Supplementation; Combination therapy (Nicotinamide + Pyruvate)
	Improvement in RNFL thickness	EGCG; Forskolin

The field of glaucoma management is undergoing a profound transformation, driven by a series of innovative treatments that offer new hope in the fight against this insidious thief of sight. While the main focus of current therapies is on reducing IOP to protect the optic nerve and preserve vision, the evolution of treatment strategies goes far beyond traditional pharmacotherapy. Traditional medications, such as β-blockers, carbonic anhydrase inhibitors, prostaglandin analogs, alpha-agonists, and cholinergic agonists, continue to play an essential role in managing IOP. However, the advent of new IOP-lowering drugs, including rho-kinase inhibitors and nitric oxide-donating prostaglandin analogs, offers promising avenues for more effective pressure control. Additionally, the development of fixed combinations and dual-mechanism therapies provides innovative strategies for addressing IOP variability in diverse patient populations. Beyond the emphasis on IOP reduction, there is a paradigm shift toward neuroprotective strategies in glaucoma treatment. Recognizing that glaucoma damage extends beyond IOP elevation, researchers are exploring ways to promote cell survival and counteract optic nerve degeneration. Approaches targeting excitotoxicity and oxidative stress, as well as modulating immune responses, are emerging to mitigate neurodegenerative processes and preserve visual function.

However, the limitations of existing treatments should not be overlooked. Long-term use of topical medications, particularly those containing preservatives, can lead to ocular surface damages, corneal toxicity, and chronic irritation, resulting in reduced patient compliance and treatment efficacy. Surgical interventions, while often necessary, are accompanied by potential complications such as scarring and unpredictable post-operative outcomes. Additionally, the complexity of multi-medication regimens remains a significant barrier to patient compliance, with many patients struggling to maintain consistent treatment, which can ultimately exacerbate disease progression. Given these challenges, the future of glaucoma management must involve a more comprehensive approach that integrates neuroprotection and IOP control, while minimizing adverse effects and improving patient compliance. Emerging therapeutic strategies represent a promising evolution toward more effective treatments that enhance patient compliance. Further research is essential to refine these approaches and ensure their long-term clinical benefits. Ultimately, the evolution of glaucoma treatments must reflect the complexity of the disease itself. A truly effective strategy will not only preserve vision by reducing IOP but also actively protect the optic nerve from irreversible damage. The next generation of therapies must strike a balance between efficacy and tolerability, enabling patients to maintain both their sight and their quality of life. As our understanding of glaucoma increases, so must our efforts to develop treatments that address its full pathophysiological spectrum.

Personalized medicine is becoming increasingly important, with genetic knowledge and biomarkers allowing clinicians to tailor interventions to each individual patient, optimizing treatment efficacy and minimizing unnecessary interventions. Machine learning algorithms have the potential to decode complex genetic pathways and identify high-risk individuals, facilitating proactive, and targeted care. As treatment guidelines evolve, extended drug delivery mechanisms and sustained-release formulations promise increased therapeutic efficacy and improved patient compliance. Moreover, advancements in imaging and biomarker discovery are driving research into precision medicine for glaucoma, enabling early detection and intervention. The convergence of innovative therapies, personalized approaches, and advanced diagnostics signals a new era in glaucoma management. By addressing the multifactorial nature of the disease and utilizing advanced interventions, clinicians are poised to transform the trajectory of glaucoma, potentially saving countless individuals from the misery of vision loss. This approach not only preserves vision but also enhances the quality of life for those affected by glaucoma.

Looking ahead, non-IOP lowering treatments will likely play a pivotal role in managing glaucoma. Factors beyond IOP, such as systemic vascular health, genetics, and lifestyle, significantly influence glaucoma progression. Impaired ocular blood flow, often linked to systemic conditions like hypertension and diabetes, can worsen optic nerve damage independently of IOP levels. Genetic variants related to neurodegeneration and apoptosis further increase susceptibility to the disease, while lifestyle factors such as obesity, smoking, and physical inactivity contribute through inflammation and oxidative stress. Additionally, cerebrospinal fluid pressure (CSFP) has been identified as an important factor, with lower CSFP associated with optic nerve damage. These findings underscore the need for a comprehensive approach that incorporates both IOP and CSFP management.

Non-IOP lowering strategies to improve ocular blood flow regulation, such as through the use of carbonic anhydrase inhibitors, calcium channel blockers, and the inhibition of astrocyte activation, offer promising therapeutic avenues. Experimental approaches such as nitric oxide-2 synthase inhibition, matrix metalloproteinase-9 suppression, and upregulation of heat shock proteins could prevent glaucomatous optic neuropathy. Additionally, reducing oxidative stress, particularly at the mitochondrial level, using antioxidants like ginkgo, polyphenolic flavonoids, and anthocyanosides, holds potential in mitigating further damage. Pre-clinical and clinical studies are necessary to confirm the efficacy of these treatments and establish their role in glaucoma management. By integrating these emerging therapies with traditional approaches, clinicians may transform the trajectory of glaucoma, preserving vision and improving the quality of life for those affected by the disease. Despite significant advancements, research on glaucoma treatment remains incomplete. Numerous innovative therapies are under investigation, with ongoing clinical trials assessing their potential efficacy. Glaucoma is extensively studied, encompassing diverse aspects ranging from its pathophysiology to novel therapeutic strategies. Currently, 163 interventional clinical trials are in progress, reflecting substantial efforts to better understand this disease and develop more effective treatments (clinicaltrials.gov). Interestingly, among the ongoing clinical trails, lowering the IOP remains the primary focus. This extensive research underscores the importance of glaucoma as a major public health concern and highlights the need to explore new therapeutic approaches, particularly in neuroprotection and IOP reduction, to enhance patient quality of life. Despite the large number of ongoing clinical trials, several promising therapies have yet to be tested in humans, emphasizing the critical need for continued research to bridge this gap and translate emerging treatments into clinical practice (Table 2).

**Table 2:** The first molecule in the therapeutic strategy is the NO-donating prostaglandin analog.

**Expected effect**	**Therapeutic strategy**	**Outcome**
Lower IOP	NO-donating prostaglandin analog	NCX 470 ophthalmic solution (0.1%) shows promising potential for managing IOP in glaucoma patients, with ongoing Phase 3b trial evaluating its effects on AH dynamics
	FC Rho-kinase inhibitor/latanoprost	PG324 (Netarsudil/Latanoprost) was non-inferior to Ganfort (Bimatoprost/Timolol) in reducing IOP
		PG324 provided superior IOP reduction compared to AR-13324 (Netarsudil) and Latanoprost
		PG324 demonstrated superior efficacy in sustained IOP reduction compared to AR-13324 and Latanoprost, with additional long-term safety data
		3-month study: PG324 showed superior efficacy in sustained IOP reduction compared to AR-13324 and Latanoprost
		Switching to Netarsudil/Latanoprost from various latanoprost-based regimens effectively lowered IOP in glaucoma and ocular hypertension patients, demonstrating its efficacy as a replacement therapy
	Cannabinoids	PEA supplementation improved RGC, but had no significant effects on IOP intraocular pressure, visual acuity, central corneal thickness, ganglion cell complex
		Results have not been reported yet
	Adenosine	Trabodenoson demonstrated significant potential in reducing IOP by 6-7 mmHg in Phase 2 trials, Phase 3 trials encountered setbacks due to incorrect dosages and regimens
		FC of trabodenoson and latanoprost resulted in significant IOP reduction through synergistic mechanisms
		PBF-677 in healthy volunteers, with close monitoring of side effects, safety and tolerability
		INO-8875 eye drops for IOP reduction in adults: safety, tolerability and efficacy
Neuroprotection / Neuroregeneration	Mitochondria and oxidative stress	Evaluation of the effects of CoQun (Coenzyme Q10 and Vitamin E)
		Evaluation of the effects of CoQun
	Immune response	Safety and efficacy of topical infliximab eye drops in preventing post-surgical glaucoma in patients undergoing their first penetrating keratoplasty
	Nicotinamide	Efficacy of nicotinamide supplementation in slowing visual field progression
		Efficacy of nicotinamide supplementation in slowing visual field progression
		Efficacy on RGC functions
		Improvement of the retinal vascularization after short-term treatment
		Investigating the combination of nicotinamide and pyruvate
		Efficacy of nicotinamide in slowing visual field loss
		Investigating the combination of citicoline and nicotinamide oral solution on short term improvement in inner retinal function, bioelectrical activity of the visual cortex and visual function
	Statins	Rosuvastatin could improve endothelial function, withdrawn
	EGCG	Potential benefits for inner retinal function
	Forskolin	Investigating RNFL modifications
		Effects of KRONEK on IOP Patients under maximum tolerated medical therapy, withdrawn
		Effects of association of forskolin with Rutin and vitamins B on IOP in Patients under treatment with either beta-blockers or prostaglandins eye drop, withdrawn

Future glaucoma research should make significant progress by focusing on several critical areas, particularly in the fields of early diagnosis, personalized treatment and innovative therapeutic delivery systems. One of the most promising avenues is the development of AI-powered diagnostic tools and continuous IOP monitoring systems, such as smart contact lenses. These lenses can facilitate real-time IOP monitoring, which is crucial for the early detection of glaucoma. In addition, the integration of nanotechnology into contact lenses has opened up new options for drug delivery systems, enabling sustained release of glaucoma drugs. These advances not only improve drug bioavailability, but also reduce the frequency of administration, thereby improving patient compliance. The use of smart contact lenses capable of releasing drugs in response to specific stimuli, such as light, represents a new approach to personalized treatment. This technology could enable glaucoma to be managed more effectively by tailoring treatment to the individual needs.

In addition to improving diagnostic and therapeutic approaches, future research should also explore the genetic and epigenetic factors that influence glaucoma. Understanding the molecular pathways involved in optic nerve degeneration may reveal new therapeutic targets. In addition, studying the role of the microbiome in glaucoma could lead to a better understanding of the disease pathophysiology and potential treatment strategies. The treatment of side effects associated with glaucoma drugs is another critical area for future research. The development of alternatives to the toxic preservatives commonly found in eye drops is essential to improve patient comfort and adherence to treatment regimens. In addition, the combination of therapies to mitigate long-term complications, such as drug resistance, is essential to improve treatment efficacy.

Telemedicine solutions also represent a significant opportunity to improve accessibility to glaucoma care worldwide. By leveraging technology to facilitate remote monitoring and consultations, healthcare providers can reach underserved populations, reducing the burden of this disease. Finally, ensuring the affordability and long-term safety of emerging therapies is essential if they are to be successfully implemented in clinical practice. As new technologies and treatments are developed, thorough assessments of their cost-effectiveness and safety profile are essential to ensure that they can be widely adopted without compromising patient care.

In conclusion, the future of glaucoma research should focus on integrating advanced diagnostic tools, personalized treatment strategies and innovative drug delivery systems, while addressing the challenges of accessibility, affordability and safety. These efforts will help improve outcomes for patients suffering from this debilitating disease.

## References

[1] Weinreb RN Aung T Medeiros FA. The pathophysiology and treatment of glaucoma: a review. JAMA. (2014) 311:1901. 10.1001/jama.2014.3192 24825645 PMC4523637

[2] Khazaeni B Zeppieri M Khazaeni L. Acute Angle-Closure Glaucoma. StatPearls, Treasure Island (FL): StatPearls Publishing. (2024). 28613607

[3] Gupta D Chen PP. Glaucoma. Am Fam Physician. (2016) 93:668–74. 27175839

[4] Gosling D Meyer JJ. Normal Tension Glaucoma. StatPearls, Treasure Island (FL): StatPearls Publishing. (2023). 35015402

[5] Drance S Anderson DR Schulzer M. Risk factors for progression of visual field abnormalities in normal-tension glaucoma. Am J Ophthalmol. (2001) 131:699–708. 10.1016/S0002-9394(01)00964-3 11384564

[6] Congdon NG Youlin Q Quigley H Hung PT Wang TH Ho TC. Biometry and primary angle-closure glaucoma among Chinese, white, and black populations. Ophthalmology. (1997) 104:1489–95. 10.1016/S0161-6420(97)30112-2 9307646

[7] Dietze J Blair K Havens SJ. Glaucoma. StatPearls, Treasure Island (FL): StatPearls Publishing. (2023).

[8] Phulke S Kaushik S Kaur S Pandav S. Steroid-induced glaucoma: an avoidable irreversible blindness. J Curr Glaucoma Pract. (2017) 11:67–72. 10.5005/jp-journals-10028-1226 28924342 PMC5577123

[9] Roberti G Oddone F Agnifili L Katsanos A Michelessi M Mastropasqua L. Steroid-induced glaucoma: Epidemiology, pathophysiology, and clinical management. Surv Ophthalmol. (2020) 65:458–72. 10.1016/j.survophthal.2020.01.002 32057761

[10] Palagani P Nair SKK. MRA clinical study of pseudoexfoliation in a tertiary eye care centre. J Evid Based Med Healthc. (2017) 4:5707–10. 10.18410/jebmh/2017/1146

[11] Tekin K Inanc M Elgin U. Monitoring and management of the patient with pseudoexfoliation syndrome: current perspectives. Clin Ophthalmol. (2019) 13:453–64. 10.2147/OPTH.S181444 30880906 PMC6402616

[12] Yibekal BT Adimassu NF Ayele FA. Pseudoexfoliation syndrome and associated factors among adults at gondar university comprehensive specialized hospital tertiary eye care and training center: a cross-sectional study. Clin Optom. (2021) 13:249–55. 10.2147/OPTO.S321716 34466050 PMC8403086

[13] Anderson DR. Normal-tension glaucoma (Low-tension glaucoma). Indian J Ophthalmol. (2011) 59:S97–101. 10.4103/0301-4738.73695 21150042 PMC3038511

[14] Fan N Wang P Tang L Liu X. Ocular blood flow and normal tension glaucoma. BioMed Res Int. (2015) 2015:308505. 10.1155/2015/308505 26558263 PMC4628977

[15] Tham Y-C Li X Wong TY Quigley HA Aung T Cheng C-Y. Global prevalence of glaucoma and projections of glaucoma burden through 2040: a systematic review and meta-analysis. Ophthalmology. (2014) 121:2081–90. 10.1016/j.ophtha.2014.05.013 24974815

[16] Kass MA Heuer DK Higginbotham EJ Johnson CA Keltner JL Miller JP. The ocular hypertension treatment study: a randomized trial determines that topical ocular hypotensive medication delays or prevents the onset of primary open-angle glaucoma. Arch Ophthalmol. (2002) 120:701–13. 10.1001/archopht.120.6.701 12049574

[17] The Advanced Glaucoma Intervention Study (AGIS): 7. The relationship between control of intraocular pressure and visual field deterioration.The AGIS Investigators. Am J Ophthalmol. (2000) 130:429–40. 10.1016/S0002-9394(00)00538-9 11024415

[18] Heijl A Leske MC Bengtsson B Hyman L Bengtsson B Hussein M. Reduction of intraocular pressure and glaucoma progression: results from the Early Manifest Glaucoma Trial. Arch Ophthalmol Chic Ill. (2002) 120:1268–79. 10.1001/archopht.120.10.1268 12365904

[19] Cvenkel B Kolko M. Current medical therapy and future trends in the management of glaucoma treatment. J Ophthalmol. (2020) 2020:1–14. 10.1155/2020/6138132 32774906 PMC7391108

[20] Sheybani A Scott R Samuelson TW Kahook MY Bettis DI Ahmed IIK. Open-angle glaucoma: burden of illness, current therapies, and the management of nocturnal IOP variation. Ophthalmol Ther. (2019) 9:1. 10.1007/s40123-019-00222-z 31732872 PMC7054505

[21] Congdon N Azuara-Blanco A Solberg Y Traverso CE Iester M Cutolo CA. Direct selective laser trabeculoplasty in open angle glaucoma study design: a multicentre, randomised, controlled, investigator-masked trial (GLAUrious). Br J Ophthalmol. (2023) 107:62–5. 10.1136/bjophthalmol-2021-319379 34433548 PMC9763163

[22] Ang BCH Lim SY Betzler BK Wong HJ Stewart MW Dorairaj S. Recent advancements in glaucoma surgery—a review. Bioengineering. (2023) 10:1096. 10.3390/bioengineering10091096 37760198 PMC10525614

[23] Song Y Zhang H Zhang Y Tang G Wan KH Lee JWY. Minimally invasive glaucoma surgery in primary angle-closure glaucoma. Asia-Pac J Ophthalmol. (2022) 11:460–9. 10.1097/APO.0000000000000561 36179337

[24] Mercieca K Figus M. Advances in glaucoma surgery. J Clin Med. (2023) 12:828. 10.3390/jcm12030828 36769476 PMC9917966

[25] Pattabiraman PP Rinkoski T Poeschla E Proia A Challa P Rao PV. RhoA GTPase-induced ocular hypertension in a rodent model is associated with increased fibrogenic activity in the trabecular meshwork. Am J Pathol. (2015) 185:496–512. 10.1016/j.ajpath.2014.10.023 25499974 PMC4305183

[26] Wang C Dang Y Waxman S Hong Y Shah P Loewen RT. Ripasudil in a model of pigmentary glaucoma. Transl Vis Sci Technol. (2020) 9:27. 10.1167/tvst.9.10.27 33024620 PMC7521183

[27] Jayanetti V Sandhu S Lusthaus JA. The latest drugs in development that reduce intraocular pressure in ocular hypertension and glaucoma. J Exp Pharmacol. (2020) 12:539–48. 10.2147/JEP.S281187 33244278 PMC7685378

[28] Tanihara H Inoue T Yamamoto T Kuwayama Y Abe H Araie M. Phase 1 clinical trials of a selective rho kinase inhibitor, K-115. JAMA Ophthalmol. (2013) 131:1288–95. 10.1001/jamaophthalmol.2013.323 23787820

[29] Tanihara H Inoue T Yamamoto T Kuwayama Y Abe H Araie M. Phase 2 randomized clinical study of a rho kinase inhibitor, K-115, in primary open-angle glaucoma and ocular hypertension. Am J Ophthalmol. (2013) 156:731–736.e2. 10.1016/j.ajo.2013.05.016 23831221

[30] Saito H Kagami S Mishima K Mataki N Fukushima A Araie M. Long-term side effects including blepharitis leading to discontinuation of ripasudil. J Glaucoma. (2019) 28:289. 10.1097/IJG.0000000000001203 30720574

[31] Tanihara H Kakuda T Sano T Kanno T Kurihara Y. Long-term intraocular pressure-lowering effects and adverse events of ripasudil in patients with glaucoma or ocular hypertension over 24 months. Adv Ther. (2022) 39:1659–77. 10.1007/s12325-021-02023-y 35150417 PMC8989847

[32] Tanihara H Yamamoto T Aihara M Koizumi N Fukushima A Kawakita K. Long-term intraocular pressure-lowering efficacy and safety of ripasudil-brimonidine fixed-dose combination for glaucoma and ocular hypertension: a multicentre, open-label, phase 3 study. Graefes Arch Clin Exp Ophthalmol. (2024) 262:2579–91. 10.1007/s00417-024-06388-y 38430227 PMC11271374

[33] Namekata K Noro T Nishijima E Sotozono A Guo X Harada C. Drug combination of topical ripasudil and brimonidine enhances neuroprotection in a mouse model of optic nerve injury. J Pharmacol Sci. (2024) 154:326–33. 10.1016/j.jphs.2024.02.011 38485351

[34] Futakuchi A Morimoto T Ikeda Y Tanihara H Inoue T. Intraocular pressure-lowering effects of ripasudil in uveitic glaucoma, exfoliation glaucoma, and steroid-induced glaucoma patients: ROCK-S, a multicentre historical cohort study. Sci Rep. (2020) 10:10308. 10.1038/s41598-020-66928-4 32587304 PMC7316751

[35] Nagpal H Kaur M. Topical ripasudil as first line treatment for ocular hypertension in uveitis cases: an analytic study. J Clin Diagn Res. (2021) 15:1–3. 10.7860/JCDR/2021/48878.14851

[36] Liu L-C Chen Y-H Lu D-W. The application of rho kinase inhibitors in the management of glaucoma. Int J Mol Sci. (2024) 25:5576. 10.3390/ijms25115576 38891764 PMC11171673

[37] Freiberg JC Spreckelsen A von Kolko M Azuara-Blanco A Virgili G. Rho kinase inhibitor for primary open-angle glaucoma and ocular hypertension. Cochrane Database Syst Rev. (2022) 2022:CD013817. 10.1002/14651858.CD013817.pub2 35686679 PMC9185806

[38] Asrani S Robin AL Serle JB Lewis RA Usner DW Kopczynski CC. Netarsudil/latanoprost fixed-dose combination for elevated intraocular pressure: three-month data from a randomized phase 3 trial. Am J Ophthalmol. (2019) 207:248–57. 10.1016/j.ajo.2019.06.016 31229466

[39] Tanihara H Inatani M Honjo M Tokushige H Azuma J Araie M. Intraocular pressure–lowering effects and safety of topical administration of a selective ROCK inhibitor, SNJ-1656, in healthy volunteers. Arch Ophthalmol. (2008) 126:309–15. 10.1001/archophthalmol.2007.76 18332309

[40] Kopczynski C Novack GD Swearingen D van Haarlem T. Ocular hypotensive efficacy, safety and systemic absorption of AR-12286 ophthalmic solution in normal volunteers. Br J Ophthalmol. (2013) 97:567–72. 10.1136/bjophthalmol-2012-302466 23435190

[41] Van de Velde S Van Bergen T Sijnave D Hollanders K Castermans K Defert O. AMA0076, a novel, locally acting rho kinase inhibitor, potently lowers intraocular pressure in New Zealand white rabbits with minimal hyperemia. Invest Ophthalmol Vis Sci. (2014) 55:1006–16. 10.1167/iovs.13-13157 24474276

[42] Hsu C-R Chen Y-H Liu C-P Chen C-H Huang K-K Huang J-W. A highly selective rho-kinase inhibitor (ITRI-E-212) potentially treats glaucoma upon topical administration with low incidence of ocular hyperemia. Invest Ophthalmol Vis Sci. (2019) 60:624–33. 10.1167/iovs.18-25252 30735565

[43] Cavet ME Vittitow JL Impagnatiello F Ongini E Bastia E. Nitric oxide (NO): an emerging target for the treatment of glaucoma. Investig Opthalmology Vis Sci. (2014) 55:5005. 10.1167/iovs.14-14515 25125670

[44] Weinreb RN Liebmann JM Martin KR Kaufman PL Vittitow JL. Latanoprostene bunod 0.024% in subjects with open-angle glaucoma or ocular hypertension: pooled phase 3 study findings. J Glaucoma. (2018) 27:7–15. 10.1097/IJG.0000000000000831 29194198 PMC7654727

[45] Kaufman PL. Latanoprostene bunod ophthalmic solution 0.024% for IOP lowering in glaucoma and ocular hypertension. Expert Opin Pharmacother. (2017) 18:433–44. 10.1080/14656566.2017.1293654 28234563

[46] Eyewire+ n.d. Aerie Pharmaceuticals Receives FDA Approval of Rocklatan for Reduction of IOP. Available online at: https://eyewire.news/news/aerie-pharmaceuticals-receives-fda-approval-of-rocklatan-for-reduction-of-iop (accessed June 19, 2024).

[47] Walters TR Ahmed IIK Lewis RA Usner DW Lopez J Kopczynski CC. Once-daily netarsudil/latanoprost fixed-dose combination for elevated intraocular pressure in the randomized phase 3 MERCURY-2 study. Ophthalmol Glaucoma. (2019) 2:280–9. 10.1016/j.ogla.2019.03.007 32672669

[48] Passani A Posarelli C Sframeli AT Perciballi L Pellegrini M Guidi G. Cannabinoids in glaucoma patients: the never-ending story. J Clin Med. (2020) 9:3978. 10.3390/jcm9123978 33302608 PMC7763320

[49] Marijuana As Medicine? The Science Beyond the Controversy. Washington, DC: National Academies Press (2000). 25077214

[50] Whiting PF Wolff RF Deshpande S Di Nisio M Duffy S Hernandez AV. Cannabinoids for medical use: a systematic review and meta-analysis. JAMA. (2015) 313:2456. 10.1001/jama.2015.6358 26103030

[51] Taskar PS Patil A Lakhani P Ashour E Gul W ElSohly MA. Δ ^9^ -Tetrahydrocannabinol derivative-loaded nanoformulation lowers intraocular pressure in normotensive rabbits. Transl Vis Sci Technol. (2019) 8:15. 10.1167/tvst.8.5.15 31588378 PMC6753841

[52] Rapino C Tortolani D Scipioni L Maccarrone M. Neuroprotection by (endo)Cannabinoids in glaucoma and retinal neurodegenerative diseases. Curr Neuropharmacol. (2018) 16:959–70. 10.2174/1570159X15666170724104305 28738764 PMC6120105

[53] Lopez MJ Nataneli N. Cannabis Use for Glaucoma and Associated Pain. StatPearls, Treasure Island (FL): StatPearls Publishing (2024). 34283478

[54] Lucas A Thirion A Schwan R Krieg J Angioi-Duprez K Laprevote V. Association between increased retinal background noise and co-occurrent regular cannabis and alcohol use. Prog Neuropsychopharmacol Biol Psychiatry. (2019) 89:335–40. 10.1016/j.pnpbp.2018.10.002 30292729

[55] Schwitzer T Henrion M-L Sarre D Albuisson E Angioi-Duprez K Giersch A. Spatial localization of retinal anomalies in regular cannabis users: the relevance of the multifocal electroretinogram. Schizophr Res. (2020) 219:56–61. 10.1016/j.schres.2019.01.013 30696610

[56] Schwitzer T Schwan R Albuisson E Giersch A Lalanne L Angioi-Duprez K. Association between regular cannabis use and ganglion cell dysfunction. JAMA Ophthalmol. (2017) 135:54–60. 10.1001/jamaophthalmol.2016.4761 27930757

[57] Joshi N Mariam H Kamath A. Cannabinoids for the treatment of glaucoma: a review. Med Cannabis Cannabinoids. (2024) 7:183–92. 10.1159/000541461 39474241 PMC11521503

[58] Lindner T Schmidl D Peschorn L Pai V Popa-Cherecheanu A Chua J. Therapeutic potential of cannabinoids in glaucoma. Pharmaceuticals. (2023) 16:1149. 10.3390/ph16081149 37631064 PMC10460067

[59] Bondok M Nguyen AX-L Lando L Wu AY. Adverse ocular impact and emerging therapeutic potential of cannabis and cannabinoids: a narrative review. Clin Ophthalmol Auckl NZ. (2024) 18:3529–56. 10.2147/OPTH.S501494 39629058 PMC11613704

[60] Bergman Z Douglas J Wang J Idowu O Kaleem M. Attitudes and perceptions toward the use of medical marijuana by glaucoma specialists. J Glaucoma. (2022) 31:67–71. 10.1097/IJG.0000000000001966 35085162

[61] Yakobashvili D Shah R Oydanich M Khouri AS. Public perception of marijuana use for the treatment of glaucoma. J Glaucoma. (2023) 32:e106–8. 10.1097/IJG.0000000000002203 36897646

[62] Weldy EW Stanley J Koduri VA McCourt EA Patnaik JL Kahook MY. Perceptions of marijuana use for glaucoma from patients, cannabis retailers, and glaucoma specialists. Ophthalmol Glaucoma. (2020) 3:453–9. 10.1016/j.ogla.2020.06.009 32782211

[63] Pietrucha-Dutczak M Amadio M Govoni S Lewin-Kowalik J Smedowski A. The role of endogenous neuroprotective mechanisms in the prevention of retinal ganglion cells degeneration. Front Neurosci. (2018) 12:834. 10.3389/fnins.2018.00834 30524222 PMC6262299

[64] Rafuse P Buys YM. Medical use of cannabis for glaucoma. Can J Ophthalmol J Can Ophtalmol. (2019) 54:7–8. 10.1016/j.jcjo.2018.11.001 30851777

[65] Pescosolido N Gatto V Stefanucci A Rusciano D. Oral treatment with the melatonin agonist agomelatine lowers the intraocular pressure of glaucoma patients. Ophthalmic Physiol Opt. (2015) 35:201–5. 10.1111/opo.12189 25600174

[66] Alkozi HA Navarro G Franco R Pintor J. Melatonin and the control of intraocular pressure. Prog Retin Eye Res. (2020) 75:100798. 10.1016/j.preteyeres.2019.100798 31560946

[67] Li K-L Shan S-W Lin F-Y Ling C-Y Wong N-W Li H-L. Regulation of aqueous humor secretion by melatonin in porcine ciliary epithelium. Int J Mol Sci. (2023) 24:5789. 10.3390/ijms24065789 36982863 PMC10051954

[68] Tosini G Iuvone M Boatright JH. Is the melatonin receptor type 1 involved in the pathogenesis of glaucoma? J Glaucoma. (2013) 22 Suppl 5:S49–50. 10.1097/IJG.0b013e3182934bb4 23733129 PMC4049348

[69] Morgan PJ Barrett P Howell HE Helliwell R. Melatonin receptors: localization, molecular pharmacology and physiological significance. Neurochem Int. (1994) 24:101–46. 10.1016/0197-0186(94)90100-7 8161940

[70] Wiechmann AF Wirsig-Wiechmann CR. Melatonin receptor mRNA and protein expression in Xenopus laevis nonpigmented ciliary epithelial cells. Exp Eye Res. (2001) 73:617–23. 10.1006/exer.2001.1073 11747362

[71] Martínez-Águila A Fonseca B Pérez de Lara MJ Pintor J. Effect of melatonin and 5-methoxycarbonylamino-n-acetyltryptamine on the intraocular pressure of normal and glaucomatous mice. J Pharmacol Exp Ther. (2016) 357:293–9. 10.1124/jpet.115.231456 26941171

[72] Dal Monte M Cammalleri M Pezzino S Corsaro R Pescosolido N Bagnoli P. Hypotensive effect of nanomicellar formulation of melatonin and agomelatine in a rat model: significance for glaucoma therapy. Diagnostics. (2020) 10:138. 10.3390/diagnostics10030138 32138160 PMC7151109

[73] Belforte NA Moreno MC de Zavalía N Sande PH Chianelli MS Keller Sarmiento MI. Melatonin: a novel neuroprotectant for the treatment of glaucoma. J Pineal Res. (2010) 48:353–64. 10.1111/j.1600-079X.2010.00762.x 20374442

[74] Hu C Feng Y Huang G Cui K Fan M Xiang W. Melatonin prevents EAAC1 deletion-induced retinal ganglion cell degeneration by inhibiting apoptosis and senescence. J Pineal Res. (2024) 76:e12916. 10.1111/jpi.12916 37786968

[75] Agorastos A Huber CG. The role of melatonin in glaucoma: implications concerning pathophysiological relevance and therapeutic potential. J Pineal Res. (2011) 50:1–7. 10.1111/j.1600-079X.2010.00816.x 21073517

[76] Vincent L Cohen W Delagrange P Boutin JA Nosjean O. Molecular and cellular pharmacological properties of 5-methoxycarbonylamino-N-acetyltryptamine (MCA-NAT): a nonspecific MT3 ligand. J Pineal Res. (2010) 48:222–9. 10.1111/j.1600-079X.2010.00746.x 20210850

[77] Rusciano D Russo C. The therapeutic trip of melatonin eye drops: from the ocular surface to the retina. Pharm Basel Switz. (2024) 17:441. 10.3390/ph17040441 38675402 PMC11054783

[78] Romeo A Kazsoki A Musumeci T Zelkó R. A clinical, pharmacological, and formulation evaluation of melatonin in the treatment of ocular disorders-a systematic review. Int J Mol Sci. (2024) 25:3999. 10.3390/ijms25073999 38612812 PMC11011996

[79] Alkozi H Sánchez-Naves J de Lara MJP Carracedo G Fonseca B Martinez-Aguila A. Elevated intraocular pressure increases melatonin levels in the aqueous humour. Acta Ophthalmol. (2017) 95:e185–9. 10.1111/aos.13253 27595784

[80] Crooke A Huete-Toral F Martínez-Águila A Martín-Gil A Pintor J. Involvement of carbonic anhydrases in the ocular hypotensive effect of melatonin analogue 5-MCA-NAT. J Pineal Res. (2012) 52:265–70. 10.1111/j.1600-079X.2011.00938.x 22107075

[81] Head KA. Natural therapies for ocular disorders, part two: cataracts and glaucoma. Altern Med Rev J Clin Ther. (2001) 6:141–66. 11302779

[82] Ou S-C Bai K-J Cheng W-H Chen J-Y Lin C-H Wen H-C. TGF-β Induced CTGF expression in human lung epithelial cells through ERK, ADAM17, RSK1, and C/EBPβ pathways. Int J Mol Sci. (2020) 21:9084. 10.3390/ijms21239084 33260349 PMC7731197

[83] Van Setten GB Trost A Schrödl F Kaser-Eichberger A Bogner B Van Setten M. Immunohistochemical detection of CTGF in the human eye. Curr Eye Res. (2016) 41:1571–9. 10.3109/02713683.2016.1143014 27337054

[84] Hassan MDS Razali N Abu Bakar AS Abu Hanipah NF Agarwal R. Connective tissue growth factor: Role in trabecular meshwork remodeling and intraocular pressure lowering. Exp Biol Med. (2023) 248:1425–36. 10.1177/15353702231199466 37873757 PMC10657592

[85] Dillinger AE Guter M Froemel F Weber GR Perkumas K Stamer WD. Intracameral delivery of layer-by-layer coated siRNA nanoparticles for glaucoma therapy. Small. (2018) 14:1803239. 10.1002/smll.201803239 30353713 PMC6599181

[86] Knox J Bou-Gharios G Hamill KJ Willoughby CE. MiR-18a-5p targets connective tissue growth factor expression and inhibits transforming growth factor β2-induced trabecular meshwork cell contractility. Genes. (2022) 13:1500. 10.3390/genes13081500 36011411 PMC9408287

[87] Wang W-H Deng A-J He S-G. A key role of microRNA-26a in the scar formation after glaucoma filtration surgery. Artif Cells Nanomedicine Biotechnol. (2018) 46:831–7. 10.1080/21691401.2017.1345926 28685590

[88] McDonnell F O'Brien C Wallace D. The role of epigenetics in the fibrotic processes associated with glaucoma. J Ophthalmol. (2014) 2014:750459. 10.1155/2014/750459 24800062 PMC3988735

[89] Lee EJ Han JC Park DY Cho J Kee C. Effect of connective tissue growth factor gene editing using adeno-associated virus-mediated CRISPR-Cas9 on rabbit glaucoma filtering surgery outcomes. Gene Ther. (2021) 28:277–86. 10.1038/s41434-020-0166-4 32541929

[90] Zhong Y Yang Z Huang W-C Luo X. Adenosine, adenosine receptors and glaucoma: an updated overview. Biochim Biophys Acta BBA - Gen Subj. (2013) 1830:2882–90. 10.1016/j.bbagen.2013.01.005 23328492

[91] Kvanta A Seregard S Sejersen S Kull B Fredholm BB. Localization of adenosine receptor messenger RNAs in the rat eye. Exp Eye Res. (1997) 65:595–602. 10.1006/exer.1996.0352 9367639

[92] Shearer TW Crosson CE. Adenosine A1 receptor modulation of MMP-2 secretion by trabecular meshwork cells. Invest Ophthalmol Vis Sci. (2002) 43:3016–20. 12202524

[93] Schlötzer-Schrehardt U Zenkel M Hofmann-Rummelt C Kruse FE Naumann GO. Functional significance of adenosine receptors in the eye and their dysregulation in pseudoexfoliation syndrome. Ophthalmol Z Dtsch Ophthalmol Ges. (2005) 102:1074–80. 10.1007/s00347-005-1216-4 15830199

[94] Agarwal R Agarwal P. Newer targets for modulation of intraocular pressure: focus on adenosine receptor signaling pathways. Expert Opin Ther Targets. (2014) 18:527–39. 10.1517/14728222.2014.888416 24579961

[95] Donegan RK Lieberman RL. Discovery of molecular therapeutics for glaucoma: challenges, successes, and promising directions. J Med Chem. (2016) 59:788–809. 10.1021/acs.jmedchem.5b00828 26356532 PMC5547565

[96] Qiu TG. Trabodenoson on trabecular meshwork rejuvenation: a comprehensive review of clinical data. Expert Opin Investig Drugs. (2021) 30:227–36. 10.1080/13543784.2021.1873276 33405971

[97] Wang H Deng Y Wan L Huang L. A comprehensive map of disease networks and molecular drug discoveries for glaucoma. Sci Rep. (2020) 10:9719. 10.1038/s41598-020-66350-w 32546683 PMC7298047

[98] Liu Y Pang I-H. Challenges in the development of glaucoma neuroprotection therapy. Cell Tissue Res. (2013) 353:253–60. 10.1007/s00441-013-1584-z 23474740

[99] Storgaard L Tran TL Freiberg JC Hauser AS Kolko M. Glaucoma clinical research: trends in treatment strategies and drug development. Front Med. (2021) 8:733080. 10.3389/fmed.2021.733080 34589504 PMC8473801

[100] Bouhenni R Dunmire J Sewell A Edward DP. Animal models of glaucoma. J Biomed Biotechnol. (2012) 2012:692609. 10.1155/2012/692609 22665989 PMC3364028

[101] Guymer C Wood JP Chidlow G Casson RJ. Neuroprotection in glaucoma: recent advances and clinical translation. Clin Experiment Ophthalmol. (2019) 47:88–105. 10.1111/ceo.13336 29900639

[102] Turner AJ Vander Wall R Gupta V Klistorner A Graham SL. DBA/2J mouse model for experimental glaucoma: pitfalls and problems. Clin Experiment Ophthalmol. (2017) 45:911–22. 10.1111/ceo.12992 28516453

[103] Tribble JR Hui F Quintero H El Hajji S Bell K Di Polo A. Neuroprotection in glaucoma: mechanisms beyond intraocular pressure lowering. Mol Aspects Med. (2023) 92:101193. 10.1016/j.mam.2023.101193 37331129

[104] Schmetterer L Scholl H Garhöfer G Janeschitz-Kriegl L Corvi F Sadda SR. Endpoints for clinical trials in ophthalmology. Prog Retin Eye Res. (2023) 97:101160. 10.1016/j.preteyeres.2022.101160 36599784

[105] Dong ZM Wollstein G Schuman JS. Clinical utility of optical coherence tomography in glaucoma. Invest Ophthalmol Vis Sci. (2016) 57:556. 10.1167/iovs.16-19933 27537415 PMC4991023

[106] Tsai JC. Innovative IOP-independent neuroprotection and neuroregeneration strategies in the pipeline for glaucoma. J Ophthalmol. (2020) 2020:9329310. 10.1155/2020/9329310 33014446 PMC7512103

[107] Lanza M Gironi Carnevale UA Mele L Bifani Sconocchia M Bartollino S Costagliola C. Morphological and functional evaluation of oral citicoline therapy in chronic open-angle glaucoma patients: a pilot study with a 2-year follow-up. Front Pharmacol. (2019) 10:1117. 10.3389/fphar.2019.01117 31611797 PMC6775502

[108] Bikbova G. Diabetes mellitus and retinal vein occlusion as risk factors for open angle glaucoma and neuroprotective therapies for retinal ganglion cell neuropathy. J Clin Exp Ophthalmol. (2011) 3:2. 10.4172/2155-9570.S3-002

[109] Tian K Shibata-Germanos S Pahlitzsch M Cordeiro MF. Current perspective of neuroprotection and glaucoma. Clin Ophthalmol Auckl NZ. (2015) 9:2109–18. 10.2147/OPTH.S80445 26635467 PMC4646599

[110] Vorwerk CK Gorla MSR Dreyer EB. An experimental basis for implicating excitotoxicity in glaucomatous optic neuropathy. Surv Ophthalmol. (1999) 43:S142–50. 10.1016/S0039-6257(99)00017-X 10416757

[111] Zeng Z You M Fan C Jang J Xia X. FABP5 regulates ROS-NLRP3 inflammasome in glutamate-induced retinal excitotoxic glaucomatous model. FASEB J Off Publ Fed Am Soc Exp Biol. (2025) 39:e70281. 10.1096/fj.202400435R 39792326

[112] Cheng Y Chen X Zhu G Li N Sun Y Luo S. Erigeron breviscapus: a promising medication for protecting the optic nerve in glaucoma. Planta Med. (2024) 90:992–1004. 10.1055/a-2409-2999 39303747

[113] Liu M Li H Yang R Ji D Xia X. GSK872 and necrostatin-1 protect retinal ganglion cells against necroptosis through inhibition of RIP1/RIP3/MLKL pathway in glutamate-induced retinal excitotoxic model of glaucoma. J Neuroinflammation. (2022) 19:262. 10.1186/s12974-022-02626-4 36289519 PMC9608931

[114] Fazel MF Abu IF Mohamad MHN Agarwal R Iezhitsa I Bakar NS. Philanthotoxin-343 attenuates retinal and optic nerve injury, and protects visual function in rats with N-methyl-D-aspartate-induced excitotoxicity. PLoS ONE. (2020) 15:e0236450. 10.1371/journal.pone.0236450 32706792 PMC7380593

[115] Xu K Li S Yang Q Zhou Z Fu M Yang X. MicroRNA-145-5p targeting of TRIM2 mediates the apoptosis of retinal ganglion cells via the PI3K/AKT signaling pathway in glaucoma. J Gene Med. (2021) 23:e3378. 10.1002/jgm.3378 34291866

[116] Sone K Mori A Sakamoto K Nakahara T. The role of microRNAs related to apoptosis for n-methyl-d-aspartic acid-induced neuronal cell death in the murine retina. Int J Mol Sci. (2024) 25:1106. 10.3390/ijms25021106 38256177 PMC10816001

[117] Vorwerk CK Lipton SA Zurakowski D Hyman BT Sabel BA Dreyer EB. Chronic low-dose glutamate is toxic to retinal ganglion cells. Toxicity blocked by memantine. Invest Ophthalmol Vis Sci. (1996) 37:1618–24. 8675405

[118] Casson RJ. Possible role of excitotoxicity in the pathogenesis of glaucoma. Clin Experiment Ophthalmol. (2006) 34:54–63. 10.1111/j.1442-9071.2006.01146.x 16451260

[119] Liberatore F Bucci D Mascio G Madonna M Di Pietro P Beneventano M. Permissive role for mGlu1 metabotropic glutamate receptors in excitotoxic retinal degeneration. Neuroscience. (2017) 363:142–9. 10.1016/j.neuroscience.2017.09.005 28918254

[120] Li Q Cheng Y Zhang S Sun X Wu J. TRPV4-induced Müller cell gliosis and TNF-α elevation-mediated retinal ganglion cell apoptosis in glaucomatous rats via JAK2/STAT3/NF-κB pathway. J Neuroinflam. (2021) 18:271. 10.1186/s12974-021-02315-8 34789280 PMC8596927

[121] Hu H Liu X Nie D Fang M Zhang J Zhang G. Insights into CD154-mediated pathways in ocular hypertensive glaucoma: The role of Müller cells and P2X7 in retinal neuroprotection and therapeutic potential. Cell Biol Int. (2023) 47:1441–52. 10.1002/cbin.12030 37132435

[122] Hu X Zhao G-L Xu M-X Zhou H Li F Miao Y. Interplay between Müller cells and microglia aggravates retinal inflammatory response in experimental glaucoma. J Neuroinflam. (2021) 18:303. 10.1186/s12974-021-02366-x 34952606 PMC8705189

[123] Miao Y Zhao G-L Cheng S Wang Z Yang X-L. Activation of retinal glial cells contributes to the degeneration of ganglion cells in experimental glaucoma. Prog Retin Eye Res. (2023) 93:101169. 10.1016/j.preteyeres.2023.101169 36736070

[124] Zhang ZQ Xie Z Chen SY Zhang X. Mitochondrial dysfunction in glaucomatous degeneration. Int J Ophthalmol. (2023) 16:811–23. 10.18240/ijo.2023.05.20 37206187 PMC10172101

[125] Mirzaei M Gupta VK Chitranshi N Deng L Pushpitha K Abbasi M. Retinal proteomics of experimental glaucoma model reveal intraocular pressure-induced mediators of neurodegenerative changes. J Cell Biochem. (2020) 121:4931–44. 10.1002/jcb.29822 32692886

[126] Bastola T Perkins GA Huu VAN Ju S Kim K-Y Shen Z. Administration of bicarbonate protects mitochondria, rescues retinal ganglion cells, and ameliorates visual dysfunction caused by oxidative stress. Antioxid Basel Switz. (2024) 13:743. 10.3390/antiox13060743 38929182 PMC11200884

[127] Edwards G Perkins GA Kim K-Y Kong Y Lee Y Choi S-H. Loss of AKAP1 triggers Drp1 dephosphorylation-mediated mitochondrial fission and loss in retinal ganglion cells. Cell Death Dis. (2020) 11:1–15. 10.1038/s41419-020-2456-6 32312949 PMC7170863

[128] Catalani E Brunetti K Del Quondam S Cervia D. Targeting mitochondrial dysfunction and oxidative stress to prevent the neurodegeneration of retinal ganglion cells. Antioxidants. (2023) 12:2011. 10.3390/antiox12112011 38001864 PMC10669517

[129] Lv B Chen T Xu Z Huo F Wei Y Yang X. Crocin protects retinal ganglion cells against H2O2-induced damage through the mitochondrial pathway and activation of NF-κB. Int J Mol Med. (2016) 37:225–32. 10.3892/ijmm.2015.2418 26718031

[130] Kim HJ Cha S Choi J-S Lee JY Kim KE Kim JK. scAAV2-Mediated expression of thioredoxin 2 and C3 transferase prevents retinal ganglion cell death and lowers intraocular pressure in a mouse model of glaucoma. Int J Mol Sci. (2023) 24:16253. 10.3390/ijms242216253 38003443 PMC10671512

[131] Millington-Ward S Palfi A Shortall C Finnegan LK Bargroff E Post IJM. AAV-NDI1 therapy provides significant benefit to murine and cellular models of glaucoma. Int J Mol Sci. (2024) 25:8876. 10.3390/ijms25168876 39201561 PMC11354491

[132] Ko M-L Peng P-H Hsu S-Y Chen C-F. Dietary deficiency of vitamin E aggravates retinal ganglion cell death in experimental glaucoma of rats. Curr Eye Res. (2010) 35:842–9. 10.3109/02713683.2010.489728 20795867

[133] Luo H Zhuang J Hu P Ye W Chen S Pang Y. Resveratrol delays retinal ganglion cell loss and attenuates gliosis-related inflammation from ischemia-reperfusion injury. Investig Opthalmology Vis Sci. (2018) 59:3879. 10.1167/iovs.18-23806 30073348

[134] Chen G Zeng L Yan F Liu J Qin M Wang F. Long-term oral administration of naringenin counteracts aging-related retinal degeneration via regulation of mitochondrial dynamics and autophagy. Front Pharmacol. (2022) 13:919905. 10.3389/fphar.2022.919905 35910364 PMC9330024

[135] Labkovich M Jacobs EB Bhargava S Pasquale LR Ritch R. Ginkgo biloba extract in ophthalmic and systemic disease, with a focus on normal-tension glaucoma. Asia-Pac J Ophthalmol. (2020) 9:215–25. 10.1097/APO.0000000000000279 32282348 PMC7299225

[136] Harris A Gross J Moore N Do T Huang A Gama W. The effects of antioxidants on ocular blood flow in patients with glaucoma. Acta Ophthalmol. (2018) 96:e237–e241. 10.1111/aos.13530 28772005

[137] Hurley DJ Normile C Irnaten M O'Brien C. The intertwined roles of oxidative stress and endoplasmic reticulum stress in glaucoma. Antioxidants. (2022) 11:886. 10.3390/antiox11050886 35624748 PMC9137739

[138] Iorga RE Moraru AD Costin D Munteanu-Dǎnulescu RS Brǎnişteanu DC. Current trends in targeting the oxidative stress in glaucoma (Review). Eur J Ophthalmol. (2024) 34:328–37. 10.1177/11206721231214297 37974458

[139] Chen X Liu J Chen M Zhou J Zhang Y Hu X. Green-light-triggered and self-calibrated cascade release of nitric oxide and carbon monoxide for synergistic glaucoma therapy. J Am Chem Soc. (2024) 146:30361–71. 10.1021/jacs.4c10457 39421962

[140] Ahmed E Donovan T Yujiao L Zhang Q. Mitochondrial targeted antioxidant in cerebral ischemia. J Neurol Neurosci. (2015) 06:17. 10.21767/2171-6625.100017 26937332 PMC4771373

[141] Russo R Cavaliere F Rombolà L Gliozzi M Cerulli A Nucci C. Rational basis for the development of coenzyme Q10 as a neurotherapeutic agent for retinal protection. Prog Brain Res. (2008) 173:575–82. 10.1016/S0079-6123(08)01139-4 18929135

[142] Sato K Saigusa D Kokubun T Fujioka A Feng Q Saito R. Author Correction: Reduced glutathione level in the aqueous humor of patients with primary open-angle glaucoma and normal-tension glaucoma. NPJ Aging. (2024) 10:8. 10.1038/s41514-024-00137-5 38245546 PMC10799874

[143] Bariş M Tezel G. Immunomodulation as a neuroprotective strategy for glaucoma treatment. Curr Ophthalmol Rep. (2019) 7:160–9. 10.1007/s40135-019-00212-1 31360618 PMC6662642

[144] Su W Li Z Jia Y Zhuo Y. Rapamycin is neuroprotective in a rat chronic hypertensive glaucoma model. PLoS ONE. (2014) 9:e99719. 10.1371/journal.pone.0099719 24923557 PMC4055719

[145] Bosco A Crish SD Steele MR Romero CO Inman DM Horner PJ. Early reduction of microglia activation by irradiation in a model of chronic glaucoma. PLoS ONE. (2012) 7:e43602. 10.1371/journal.pone.0043602 22952717 PMC3431380

[146] Bosco A Inman DM Steele MR Wu G Soto I Marsh-Armstrong N. Reduced retina microglial activation and improved optic nerve integrity with minocycline treatment in the DBA/2J mouse model of glaucoma. Investig Opthalmology Vis Sci. (2008) 49:1437. 10.1167/iovs.07-1337 18385061

[147] Roh M Zhang Y Murakami Y Thanos A Lee SC Vavvas DG. Etanercept, a widely used inhibitor of tumor necrosis factor-α (TNF- α), prevents retinal ganglion cell loss in a rat model of glaucoma. PLoS ONE. (2012) 7:e40065. 10.1371/journal.pone.0040065 22802951 PMC3388998

[148] Madeira MH Elvas F Boia R Gonçalves FQ Cunha RA Ambrósio AF. Adenosine A2AR blockade prevents neuroinflammation-induced death of retinal ganglion cells caused by elevated pressure. J Neuroinflammation. (2015) 12:115. 10.1186/s12974-015-0333-5 26054642 PMC4465153

[149] Fernández-Albarral JA Ramírez AI de Hoz R Matamoros JA Salobrar-García E Elvira-Hurtado L. Glaucoma: from pathogenic mechanisms to retinal glial cell response to damage. Front Cell Neurosci. (2024) 18:1354569. 10.3389/fncel.2024.1354569 38333055 PMC10850296

[150] Lozano DC Choe TE Cepurna WO Morrison JC Johnson EC. Early optic nerve head glial proliferation and jak-stat pathway activation in chronic experimental glaucoma. Invest Ophthalmol Vis Sci. (2019) 60:921–32. 10.1167/iovs.18-25700 30835784 PMC6402265

[151] Chong RS Martin KR. Glial cell interactions and glaucoma. Curr Opin Ophthalmol. (2015) 26:73–7. 10.1097/ICU.0000000000000125 25490529 PMC4323570

[152] Pitha I Kambhampati S Sharma A Sharma R McCrea L Mozzer A. Targeted microglial attenuation through dendrimer-drug conjugates improves glaucoma neuroprotection. Biomacromolecules. (2023) 24:1355–65. 10.1021/acs.biomac.2c01381 36827603 PMC10189638

[153] Reinehr S Reinhard J Gandej M Gottschalk I Stute G Faissner A. S100B immunization triggers NFκB and complement activation in an autoimmune glaucoma model. Sci Rep. (2018) 8:9821. 10.1038/s41598-018-28183-6 29959432 PMC6026137

[154] Astafurov K Elhawy E Ren L Dong CQ Igboin C Hyman L. Oral microbiome link to neurodegeneration in glaucoma. PLoS ONE. (2014) 9:e104416. 10.1371/journal.pone.0104416 25180891 PMC4152129

[155] Dong L Hu Y Zhou L Cheng X. P2X7 receptor antagonist protects retinal ganglion cells by inhibiting microglial activation in a rat chronic ocular hypertension model. Mol Med Rep. (2018) 17:2289–96. 10.3892/mmr.2017.8137 29207073 PMC5783460

[156] Narayan DS Casson RJ Ebneter A Chidlow G Grace PM Hutchinson MR. Immune priming and experimental glaucoma: the effect of prior systemic lipopolysaccharide challenge on tissue outcomes after optic nerve injury. Clin Experiment Ophthalmol. (2014) 42:539–54. 10.1111/ceo.12289 24373007

[157] Zhou H Yang R-K Li Q Li Z Wang Y-C Li S-Y. MicroRNA-146a-5p protects retinal ganglion cells through reducing neuroinflammation in experimental glaucoma. Glia. (2024) 72:2115–41. 10.1002/glia.24600 39041109

[158] Chen S Wang N Xiong S Xia X. The correlation between primary open-angle glaucoma (POAG) and gut microbiota: a pilot study towards predictive, preventive, and personalized medicine. EPMA J. (2023) 14:539–52. 10.1007/s13167-023-00336-2 37605653 PMC10439875

[159] Cueva Vargas JL Belforte N Di Polo A. The glial cell modulator ibudilast attenuates neuroinflammation and enhances retinal ganglion cell viability in glaucoma through protein kinase A signaling. Neurobiol Dis. (2016) 93:156–71. 10.1016/j.nbd.2016.05.002 27163643

[160] Howell GR Soto I Zhu X Ryan M Macalinao DG Sousa GL. Radiation treatment inhibits monocyte entry into the optic nerve head and prevents neuronal damage in a mouse model of glaucoma. J Clin Invest. (2012) 122:1246–61. 10.1172/JCI61135 22426214 PMC3314470

[161] Smith MA Plyler ES Dengler-Crish CM Meier J Crish SD. Nodes of ranvier in glaucoma. Neuroscience. (2018) 390:104–18. 10.1016/j.neuroscience.2018.08.016 30149050

[162] Kuehn MH Zeng H Alward W Kwon YH Fingert JH Sears N. T-cell profiling of glaucoma patients. Invest Ophthalmol Vis Sci. (2022) 63:942–A0411.

[163] Zeng H Kuehn MH. Increased T-regulatory cell activity protects retinal ganglion cells in glaucoma. Invest Ophthalmol Vis Sci. (2023) 64:3922.

[164] Tezel G. TNF-α signaling in glaucomatous neurodegeneration. Prog Brain Res. (2008) 173:409–21. 10.1016/S0079-6123(08)01128-X 18929124 PMC3150483

[165] Kitaoka Y Kitaoka Y Kwong JMK Ross-Cisneros FN Wang J Tsai RK. TNF-α-induced optic nerve degeneration and nuclear factor-κB p65. Investig Opthalmology Vis Sci. (2006) 47:1448. 10.1167/iovs.05-0299 16565378

[166] Yang X Hondur G Tezel G. Antioxidant treatment limits neuroinflammation in experimental glaucoma. Investig Opthalmology Vis Sci. (2016) 57:2344. 10.1167/iovs.16-19153 27127934 PMC4855827

[167] Bell K Funke S Grus FH. Autoimmunität und glaukom. Ophthalmol. (2019) 116:18–27. 10.1007/s00347-018-0658-4 29427020

[168] Gris P Tighe A Levin D Sharma R Brown A. Transcriptional regulation of scar gene expression in primary astrocytes. Glia. (2007) 55:1145–55. 10.1002/glia.20537 17597120

[169] Junier M-P. What role(s) for TGFα in the central nervous system? Prog Neurobiol. (2000) 62:443–73. 10.1016/S0301-0082(00)00017-4 10869779

[170] Pasquin S Sharma M Gauchat J-F. Ciliary neurotrophic factor (CNTF): new facets of an old molecule for treating neurodegenerative and metabolic syndrome pathologies. Cytokine Growth Factor Rev. (2015) 26:507–15. 10.1016/j.cytogfr.2015.07.007 26187860

[171] Shinozaki Y Namekata K Guo X Harada T. Glial cells as a promising therapeutic target of glaucoma: beyond the IOP. Front Ophthalmol. (2024) 3:1310226. 10.3389/fopht.2023.1310226 38983026 PMC11182302

[172] Shpak AA Guekht AB Druzhkova TA Kozlova KI Gulyaeva NV. Ciliary neurotrophic factor in patients with primary open-angle glaucoma and age-related cataract. Mol Vis. (2017) 23:799–809. 29225456 PMC5710971

[173] Balzamino BO Esposito G Marino R Keller F Micera A. NGF expression in reelin-deprived retinal cells: a potential neuroprotective. Effect Neuromolecular Med. (2015) 17:314–25. 10.1007/s12017-015-8360-z 26066836

[174] Nam M-H Stankowska DL Johnson GA Nahomi RB Pantcheva MB Nagaraj RH. Peptains block retinal ganglion cell death in animal models of ocular hypertension: implications for neuroprotection in glaucoma. Cell Death Dis. (2022) 13:1–10. 10.1038/s41419-022-05407-2 36379926 PMC9666629

[175] Gardiner SK Cull G Fortune B Wang L. Increased optic nerve head capillary blood flow in early primary open-angle glaucoma. Invest Ophthalmol Vis Sci. (2019) 60:3110–8. 10.1167/iovs.19-27389 31323681 PMC6645706

[176] Hwang JC Konduru R Zhang X Tan O Francis BA Varma R. Relationship among visual field, blood flow, and neural structure measurements in glaucoma. Invest Ophthalmol Vis Sci. (2012) 53:3020–6. 10.1167/iovs.11-8552 22447865 PMC3378085

[177] Venkataraman ST Flanagan JG Hudson C. Vascular reactivity of optic nerve head and retinal blood vessels in glaucoma–a review. Microcirc N Y N. (1994) 17:568–81. 10.1111/j.1549-8719.2010.00045.x 21040122

[178] Chong RS Osborne A Conceição R Martin KR. Platelet-derived growth factor preserves retinal synapses in a rat model of ocular hypertension. Invest Ophthalmol Vis Sci. (2016) 57:842–52. 10.1167/iovs.15-17864 26934142

[179] Takahama S Adetunji MO Zhao T Chen S Li W Tomarev SI. Retinal astrocytes and GABAergic wide-field amacrine cells express PDGFRα: connection to retinal ganglion cell neuroprotection by PDGF-AA. Invest Ophthalmol Vis Sci. (2017) 58:4703–11. 10.1167/iovs.21783 28910446 PMC5606213

[180] Thanos CG Bell WJ O'Rourke P Kauper K Sherman S Stabila P. Sustained secretion of ciliary neurotrophic factor to the vitreous, using the encapsulated cell therapy-based NT-501 intraocular device. Tissue Eng. (2004) 10:1617–22. 10.1089/ten.2004.10.1617 15684670

[181] Sieving PA Caruso RC Tao W Coleman HR Thompson DJS Fullmer KR. Ciliary neurotrophic factor (CNTF) for human retinal degeneration: Phase I trial of CNTF delivered by encapsulated cell intraocular implants. Proc Natl Acad Sci. (2006) 103:3896–901. 10.1073/pnas.0600236103 16505355 PMC1383495

[182] Tao W. Application of encapsulated cell technology for retinal degenerative diseases. Expert Opin Biol Ther. (2006) 6:717–26. 10.1517/14712598.6.7.717 16805711

[183] Birch DG Weleber RG Duncan JL Jaffe GJ Tao W. Randomized trial of ciliary neurotrophic factor delivered by encapsulated cell intraocular implants for retinitis pigmentosa. Am J Ophthalmol. (2013) 156:283–292.e1. 10.1016/j.ajo.2013.03.021 23668681 PMC4111936

[184] Zhang K Hopkins JJ Heier JS Birch DG Halperin LS Albini TA. Ciliary neurotrophic factor delivered by encapsulated cell intraocular implants for treatment of geographic atrophy in age-related macular degeneration. Proc Natl Acad Sci. (2011) 108:6241–5. 10.1073/pnas.1018987108 21444807 PMC3076847

[185] Goldberg JL. CNTF Cell implants for glaucoma: a phase I study. clinicaltrials.gov (2016).

[186] Williams PA Harder JM Cardozo BH Foxworth NE John SWM. Nicotinamide treatment robustly protects from inherited mouse glaucoma. Commun Integr Biol. (2018) 11:e1356956. 10.1080/19420889.2017.1356956 29497468 PMC5824969

[187] Zhang X Zhang N Chrenek MA Girardot PE Wang J Sellers JT. Systemic treatment with nicotinamide riboside is protective in two mouse models of retinal ganglion cell damage. Pharmaceutics. (2021) 13:893. 10.3390/pharmaceutics13060893 34208613 PMC8235058

[188] Jung KI Han J-S Park CK. Neuroprotective effects of nicotinamide (Vitamin B3) on neurodegeneration in diabetic rat retinas. Nutrients. (2022) 14:1162. 10.3390/nu14061162 35334819 PMC8950738

[189] Kuo C-Y Liu CJ-L. Neuroprotection in glaucoma: basic aspects and clinical relevance. J Pers Med. (2022) 12:1884. 10.3390/jpm12111884 36579616 PMC9697907

[190] Petriti B Williams PA Lascaratos G Chau K-Y Garway-Heath DF. Neuroprotection in glaucoma: NAD+/NADH redox state as a potential biomarker and therapeutic target. Cells. (2021) 10:1402. 10.3390/cells10061402 34198948 PMC8226607

[191] Tribble JR Otmani A Sun S Ellis SA Cimaglia G Vohra R. Nicotinamide provides neuroprotection in glaucoma by protecting against mitochondrial and metabolic dysfunction. Redox Biol. (2021) 43:101988. 10.1016/j.redox.2021.101988 33932867 PMC8103000

[192] Williams PA Harder JM Foxworth NE Cardozo BH Cochran KE John SWM. Nicotinamide and WLDS act together to prevent neurodegeneration in glaucoma. Front Neurosci. (2017) 11:232. 10.3389/fnins.2017.00232 28487632 PMC5403885

[193] Pappenhagen N Yin E Morgan AB Kiehlbauch CC Inman DM. Stretch stress propels glutamine dependency and glycolysis in optic nerve head astrocytes. Front Neurosci. (2022) 16:957034. 10.3389/fnins.2022.957034 35992925 PMC9389405

[194] Babighian S Gattazzo I Zanella MS Galan A D'Esposito F Musa M. Nicotinamide: bright potential in glaucoma management. Biomedicines. (2024) 12:1655. 10.3390/biomedicines12081655 39200120 PMC11352092

[195] Chou T-H Romano GL Amato R Porciatti V. Nicotinamide-rich diet in DBA/2J mice preserves retinal ganglion cell metabolic function as assessed by PERG adaptation to flicker. Nutrients. (2020) 12:1910. 10.3390/nu12071910 32605122 PMC7401244

[196] Hui F Tang J Williams PA McGuinness MB Hadoux X Casson RJ. Improvement in inner retinal function in glaucoma with nicotinamide (vitamin B3) supplementation: a crossover randomised clinical trial. Clin Exp Ophthalmol. (2020) 48:903–914. 10.1111/ceo.13818 32721104

[197] Cimaglia G Tribble JR Votruba M Williams PA Morgan JE. Oral nicotinamide provides robust, dose-dependent structural and metabolic neuroprotection of retinal ganglion cells in experimental glaucoma. Acta Neuropathol Commun. (2024) 12:137. 10.1186/s40478-024-01850-8 39180087 PMC11342512

[198] Nagaoka T Takahashi A Sato E Izumi N Hein TW Kuo L. Effect of systemic administration of simvastatin on retinal circulation. Arch Ophthalmol. (2006) 124:665–70. 10.1001/archopht.124.5.665 16682588

[199] Kwak B Mulhaupt F Myit S Mach F. Statins as a newly recognized type of immunomodulator. Nat Med. (2000) 6:1399–402. 10.1038/82219 11100127

[200] Kim M-L Sung KR Kwon J Choi GW Shin JA. Neuroprotective effect of statins in a rat model of chronic ocular hypertension. Int J Mol Sci. (2021) 22:12500. 10.3390/ijms222212500 34830387 PMC8621698

[201] Fernández-Navarro J Aldea P de Hoz R Salazar JJ Ramírez AI Rojas B. Neuroprotective effects of low-dose statins in the retinal ultrastructure of hypercholesterolemic rabbits. PLoS ONE. (2016) 11:e0154800. 10.1371/journal.pone.0154800 27144842 PMC4856380

[202] Pokrovskaya O Wallace D O'Brien C. The emerging role of statins in glaucoma pathological mechanisms and therapeutics. Open J Ophthalmol. (2014) 4:124–38. 10.4236/ojoph.2014.44021

[203] Cong L Fu S Zhang J Zhao J Zhang Y. Effects of atorvastatin on porcine aqueous humour outflow and trabecular meshwork cells. Exp Ther Med. (2018) 15:210–6. 10.3892/etm.2017.5353 29250149 PMC5729697

[204] Choi GW Kim M-L Sung KR. Modulation of TRPV4-mediated TNF-α expression in Müller glia and subsequent RGC apoptosis by statins. Exp Eye Res. (2024) 239:109781. 10.1016/j.exer.2024.109781 38184223

[205] Song X-Y Chen Y-Y Liu W-T Cong L Zhang J-L Zhang Y. Atorvastatin reduces IOP in ocular hypertension *in vivo* and suppresses ECM in trabecular meshwork perhaps via FGD4. Int J Mol Med. (2022) 49:1–10. 10.3892/ijmm.2022.5132 35417030 PMC9015665

[206] Yilmaz N Coban DT Bayindir A Erol MK Ellidag HY Giray O. Higher serum lipids and oxidative stress in patients with normal tension glaucoma, but not pseudoexfoliative glaucoma. Bosn J Basic Med Sci. (2016) 16:21–7. 10.17305/bjbms.2016.830 26773174 PMC4765935

[207] Lee SY Paul ME Coleman AL Kitayama K Yu F Pan D. Associations between statin use and glaucoma in the all of us research program. Ophthalmol Glaucoma. (2024) 7:563–71. 10.1016/j.ogla.2024.07.008 39094953

[208] Talwar N Musch DC Stein JD. Association of daily dosage and type of statin agent with risk of open-angle glaucoma. JAMA Ophthalmol. (2017) 135:263–7. 10.1001/jamaophthalmol.2016.5406 28114645 PMC6126374

[209] Shen C Chen L Jiang L Lai TYY. Neuroprotective effect of epigallocatechin-3-gallate in a mouse model of chronic glaucoma. Neurosci Lett. (2015) 600:132–6. 10.1016/j.neulet.2015.06.002 26050640

[210] Zhang W-H Chen Y Gao L-M Cao Y-N. Neuroprotective role of epigallocatechin-3-gallate in acute glaucoma via the nuclear factor-κB signalling pathway. Exp Ther Med. (2021) 22:1235. 10.3892/etm.2021.10669 34539831 PMC8438659

[211] Herges K Millward JM Hentschel N Infante-Duarte C Aktas O Zipp F. Neuroprotective effect of combination therapy of glatiramer acetate and epigallocatechin-3-gallate in neuroinflammation. PLoS ONE. (2011) 6:e25456. 10.1371/journal.pone.0025456 22022398 PMC3192751

[212] Seong K-J Lee H-G Kook MS Ko H-M Jung J-Y Kim W-J. Epigallocatechin-3-gallate rescues LPS-impaired adult hippocampal neurogenesis through suppressing the TLR4-NF-κB signaling pathway in mice. Korean J Physiol Pharmacol. (2016) 20:41–51. 10.4196/kjpp.2016.20.1.41 26807022 PMC4722190

[213] Tang Y Wang J Wan S Luo L Qiu Y Jiang S. Epigallocatechin gallate enhances the motor neuron survival and functional recovery after brachial plexus root avulsion by regulating FIG4. Folia Neuropathol. (2019) 57:340–7. 10.5114/fn.2019.90819 32337947

[214] Khalatbary AR Khademi E. The green tea polyphenolic catechin epigallocatechin gallate and neuroprotection. Nutr Neurosci. (2020) 23:281–94. 10.1080/1028415X.2018.1500124 30043683

[215] Zhao X Liu F Jin H Li R Wang Y Zhang W. Involvement of PKCα and ERK1/2 signaling pathways in EGCG's protection against stress-induced neural injuries in Wistar rats. Neuroscience. (2017) 346:226–37. 10.1016/j.neuroscience.2017.01.025 28131624 PMC5421386

[216] Feng S Liu J Cheng B Deng A Zhang H. (–)-Epigallocatechin-3-gallate protects PC12 cells against corticosterone-induced neurotoxicity via the hedgehog signaling pathway. Exp Ther Med. (2018) 15:4284–90. 10.3892/etm.2018.5936 29731823 PMC5920970

[217] Zhao X Li R Jin H Jin H Wang Y Zhang W. Epigallocatechin-3-gallate confers protection against corticosterone-induced neuron injuries via restoring extracellular signal-regulated kinase 1/2 and phosphatidylinositol-3 kinase/protein kinase B signaling pathways. PLoS ONE. (2018) 13:e0192083. 10.1371/journal.pone.0192083 29373584 PMC5786317

[218] Xie J Jiang L Zhang T Jin Y Yang D Chen F. Neuroprotective effects of Epigallocatechin-3-gallate (EGCG) in optic nerve crush model in rats. Neurosci Lett. (2010) 479:26–30. 10.1016/j.neulet.2010.05.020 20471452

[219] Alasbahi RH Melzig MF. Forskolin and derivatives as tools for studying the role of cAMP. Pharm. (2012) 67:5–13. 10.1691/ph.2012.1642 22393824

[220] Locri F Cammalleri M Dal Monte M Rusciano D Bagnoli P. Protective efficacy of a dietary supplement based on forskolin, homotaurine, spearmint extract, and group B vitamins in a mouse model of optic nerve injury. Nutrients. (2019) 11:2931. 10.3390/nu11122931 31816880 PMC6950150

[221] Pateraki I Andersen-Ranberg J Jensen NB Wubshet SG Heskes AM Forman V. Total biosynthesis of the cyclic AMP booster forskolin from *Coleus forskohlii*. Elife. (2017) 6:e23001. 10.7554/eLife.23001 28290983 PMC5388535

[222] Wagh VD Patil PN Surana SJ Wagh KV. Forskolin: upcoming antiglaucoma molecule. J Postgrad Med. (2012) 58:199–202. 10.4103/0022-3859.101396 23023353

[223] Majeed M Nagabhushanam K Natarajan S Vaidyanathan P Karri SK Jose JA. Efficacy and safety of 1% forskolin eye drops in open angle glaucoma - An open label study. Saudi J Ophthalmol. (2015) 29:197–200. 10.1016/j.sjopt.2015.02.003 26155078 PMC4487936

[224] Vetrugno M Uva MG Russo V Iester M Ciancaglini M Brusini P. Oral administration of forskolin and rutin contributes to intraocular pressure control in primary open angle glaucoma patients under maximum tolerated medical therapy. J Ocul Pharmacol Ther. (2012) 28:536–41. 10.1089/jop.2012.0021 22731245

[225] Satriano A Laganà ML Licastro E Nucci C Bagetta G Russo R. Neuroprotective effect of a nutritional supplement containing spearmint extract, forskolin, homotaurine and group B vitamins in a mouse model of transient ocular hypertension. Biomedicines. (2023) 11:1478. 10.3390/biomedicines11051478 37239149 PMC10216629

[226] Adornetto A Rombolà L Morrone LA Nucci C Corasaniti MT Bagetta G. Natural products: evidence for neuroprotection to be exploited in glaucoma. Nutrients. (2020) 12:3158. 10.3390/nu12103158 33081127 PMC7602834

[227] Russo R Adornetto A Cavaliere F Varano GP Rusciano D Morrone LA. Intravitreal injection of forskolin, homotaurine, and L-carnosine affords neuroprotection to retinal ganglion cells following retinal ischemic injury. Mol Vis. (2015) 21:718–29. 26167113 PMC4483367

[228] Cammalleri M Dal Monte M Amato R Bagnoli P Rusciano D. A dietary combination of forskolin with homotaurine, spearmint and b vitamins protects injured retinal ganglion cells in a rodent model of hypertensive glaucoma. Nutrients. (2020) 12:1189. 10.3390/nu12041189 32340314 PMC7230514

[229] Lavik E Kuehn MH Kwon YH. Novel drug delivery systems for glaucoma. Eye Lond Engl. (2011) 25:578–86. 10.1038/eye.2011.82 21475311 PMC3171267

[230] Ichhpujani P Thakur S. iDose TR Sustained-release travoprost implant for the treatment of glaucoma. Ophthalmology. (2023) 17:4–7. 10.17925/USOR.2023.17.1.4

[231] Jumelle C Gholizadeh S Annabi N Dana R. Advances and limitations of drug delivery systems formulated as eye drops. J Control Release. (2020) 321:1. 10.1016/j.jconrel.2020.01.057 32027938 PMC7170772

[232] Cassano R Di Gioia ML Trombino S. Gel-based materials for ophthalmic drug delivery. Gels. (2021) 7:130. 10.3390/gels7030130 34563016 PMC8482217

[233] Meza-Rios A Navarro-Partida J Armendariz-Borunda J Santos A. Therapies based on nanoparticles for eye drug delivery. Ophthalmol Ther. (2020) 9:1–14. 10.1007/s40123-020-00257-7 32383107 PMC7406616

[234] Craven ER Walters T Christie WC Day DG Lewis RA Goodkin ML. 24-Month Phase I/II clinical trial of bimatoprost sustained-release implant (bimatoprost SR) in glaucoma patients. Drugs. (2020) 80:167–79. 10.1007/s40265-019-01248-0 31884564 PMC7007425

[235] Berdahl JP Sarkisian SR Ang RE Doan LV Kothe AC Usner DW. Efficacy and safety of the travoprost intraocular implant in reducing topical IOP-lowering medication burden in patients with open-angle glaucoma or ocular hypertension. Drugs. (2024) 84:83–97. 10.1007/s40265-023-01973-7 38060092 PMC10789685

[236] Sarkisian SR Ang RE Lee AM Berdahl JP Heersink SB Burden JH. Travoprost intracameral implant for open-angle glaucoma or ocular hypertension: 12-month results of a randomized, double-masked trial. Ophthalmol Ther. (2024) 13:995–1014. 10.1007/s40123-024-00898-y 38345710 PMC10912401

[237] Peral A Martinez-Aguila A Pastrana C Huete-Toral F Carpena-Torres C Carracedo G. Contact lenses as drug delivery system for glaucoma: a review. Appl Sci. (2020) 10:5151. 10.3390/app10155151

[238] Zhao L Song J Du Y Ren C Guo B Bi H. Therapeutic applications of contact lens-based drug delivery systems in ophthalmic diseases. Drug Deliv. (2023) 30:2219419. 10.1080/10717544.2023.2219419 37264930 PMC10240982

[239] Mun J Mok J won Jeong S Cho S Joo C-K Hahn SK. Drug-eluting contact lens containing cyclosporine-loaded cholesterol-hyaluronate micelles for dry eye syndrome. RSC Adv. (2019) 9:16578–85. 10.1039/C9RA02858G 35516366 PMC9064448

[240] Lee D Cho S Park HS Kwon I. Ocular drug delivery through pHEMA-hydrogel contact lenses co-loaded with lipophilic vitamins. Sci Rep. (2016) 6:34194. 10.1038/srep34194 27678247 PMC5039753

[241] Cai R Zhang L Chi H. Recent development of polymer nanomicelles in the treatment of eye diseases. Front Bioeng Biotechnol. (2023) 11:1246974. 10.3389/fbioe.2023.1246974 37600322 PMC10436511

[242] Pelusi L Mandatori D Mastropasqua L Agnifili L Allegretti M Nubile M. Innovation in the development of synthetic and natural ocular drug delivery systems for eye diseases treatment: focusing on drug-loaded ocular inserts, contacts, and intraocular lenses. Pharmaceutics. (2023) 15:625. 10.3390/pharmaceutics15020625 36839947 PMC9961328

[243] Alshammari F. Glaucoma: a review of current management, patients' adherence, direct and indirect cost, and barriers to drug delivery. Trop J Pharm Res. (2024) 23:215–21. 10.4314/tjpr.v23i1.27

[244] Miller PE Eaton JS. Medical anti-glaucoma therapy: beyond the drop. Vet Ophthalmol. (2021) 24 Suppl 1:2–15. 10.1111/vop.12843 33164328

[245] Fedorchak MV Conner IP Schuman JS Cugini A Little SR. Long term glaucoma drug delivery using a topically retained gel/microsphere eye drop. Sci Rep. (2017) 7:8639. 10.1038/s41598-017-09379-8 28819134 PMC5561248

[246] Shafiee A Bowman LM Hou E Hosseini K. Ocular pharmacokinetics of bimatoprost formulated in DuraSite compared to bimatoprost 0.03% ophthalmic solution in pigmented rabbit eyes. Clin Ophthalmol. (2013) 7:1549–56. 10.2147/OPTH.S48766 23940414 PMC3737010

[247] Brandt JD DuBiner HB Benza R Sall KN Walker GA Semba CP. Long-term safety and efficacy of a sustained-release bimatoprost ocular ring. Ophthalmology. (2017) 124:1565–6. 10.1016/j.ophtha.2017.04.022 28528010

[248] Lewis RA Christie WC Day DG Craven ER Walters T Bejanian M. Bimatoprost sustained-release implants for glaucoma therapy: 6-month results from a phase I/II clinical trial. Am J Ophthalmol. (2017) 175:137–47. 10.1016/j.ajo.2016.11.020 28012819

[249] Navratil T Garcia A Tully J Maynor B Ahmed IIK Budenz DL. Preclinical evaluation of ENV515 (travoprost) intracameral implant - clinical candidate for treatment of glaucoma targeting six-month duration of action. Invest Ophthalmol Vis Sci. (2014) 55:3548.

[250] Therapeutics E. Envisia Therapeutics Releases Interim ENV515 (Travoprost XR) Phase 2 Data Demonstrating 11-Month Duration-of-Action After a Single Dose in Patients With Glaucoma. Available online at: https://www.prnewswire.com/news-releases/envisia-therapeutics-releases-interim-env515-travoprost-xr-phase-2-data-demonstrating-11-month-duration-of-action-after-a-single-dose-in-patients-with-glaucoma-300401812.html (accessed October 22, 2024).

[251] Trevino L Navratil T Robeson R Garcia A Tully J Hunter M. Intracameral conversion of travoprost to travoprost acid in the normotensive beagle dog model. Invest Ophthalmol Vis Sci. (2014) 55:5270.

[252] Komaromy AM Koehl K Harman CD Stewart G Wolinski N Norris TN. Long-term intraocular pressure (IOP) control by means of a novel biodegradable intracameral (IC) latanoprost free acid (LFA) implant. Invest Ophthalmol Vis Sci. (2017) 58:4591.

[253] Kim J Kudisch M da Silva NRK Asada H Aya-Shibuya E Bloomer MM. Long-term intraocular pressure reduction with intracameral polycaprolactone glaucoma devices that deliver a novel anti-glaucoma agent. J Control Release. (2018) 269:45–51. 10.1016/j.jconrel.2017.11.008 29127001 PMC5748363

[254] Kim J Kudisch M Mudumba S Asada H Aya-Shibuya E Bhisitkul RB. Biocompatibility and pharmacokinetic analysis of an intracameral polycaprolactone drug delivery implant for glaucoma. Invest Ophthalmol Vis Sci. (2016) 57:4341–6. 10.1167/iovs.16-19585 27556217 PMC5015984

[255] Awwad S Mohamed Ahmed AHA Sharma G Heng JS Khaw PT Brocchini S. Principles of pharmacology in the eye. Br J Pharmacol. (2017) 174:4205–23. 10.1111/bph.14024 28865239 PMC5715579

[256] Fahmy HM Saad EAES Sabra NM El-Gohary AA Mohamed FF Gaber MH. Treatment merits of Latanoprost/Thymoquinone - Encapsulated liposome for glaucomatus rabbits. Int J Pharm. (2018) 548:597–608. 10.1016/j.ijpharm.2018.07.012 29997042

[257] pSivida Announces Phase I/II Clinical Study Evaluating Bioerodible Sustained Release Latanoprost Device in Ocular Hypertension and Glaucoma. Available online at: https://cdn.pfizer.com/pfizercom/partnering/061411_psivida_announces_phase_clinical_study.pdf (accessed October 22, 2024).

[258] Hoang DM Pham PT Bach TQ Ngo ATL Nguyen QT Phan TTK. Stem cell-based therapy for human diseases. Signal Transduct Target Ther. (2022) 7:272. 10.1038/s41392-022-01134-4 35933430 PMC9357075

[259] Sotiropulos K Kourkoutas D Almaliotis D Ploumidou K Karampatakis V. Ocular stem cells: a narrative review of current clinical trials. Int J Ophthalmol. (2022) 15:1529–37. 10.18240/ijo.2022.09.17 36124200 PMC9453397

[260] Rizkiawan DE Evelyn M Tjandra KC Setiawan B. Utilization of modified induced pluripotent stem cells as the advance therapy of glaucoma: a systematic review. Clin Ophthalmol Auckl NZ. (2022) 16:2851–9. 10.2147/OPTH.S372114 36061629 PMC9439642

[261] Du Y Yun H Yang E Schuman JS. Stem cells from trabecular meshwork home to TM tissue in vivo. Invest Ophthalmol Vis Sci. (2013) 54:1450–9. 10.1167/iovs.12-11056 23341019 PMC4604717

[262] Manuguerra-GagnÉ R Boulos PR Ammar A Leblond FA Krosl G Pichette V. Transplantation of mesenchymal stem cells promotes tissue regeneration in a glaucoma model through laser-induced paracrine factor secretion and progenitor cell recruitment. Stem Cells. (2013) 31:1136–48. 10.1002/stem.1364 23495088

[263] Braunger BM Ademoglu B Koschade SE Fuchshofer R Gabelt BT Kiland JA. Identification of adult stem cells in schwalbe's line region of the primate eye. Invest Ophthalmol Vis Sci. (2014) 55:7499–507. 10.1167/iovs.14-14872 25324280 PMC4575086

[264] Roubeix C Godefroy D Mias C Sapienza A Riancho L Degardin J. Intraocular pressure reduction and neuroprotection conferred by bone marrow-derived mesenchymal stem cells in an animal model of glaucoma. Stem Cell Res Ther. (2015) 6:177. 10.1186/s13287-015-0168-0 26377305 PMC4574127

[265] Zhou Y Xia X Yang E Wang Y Marra KG Ethier CR. Adipose-derived stem cells integrate into trabecular meshwork with glaucoma treatment potential. FASEB J. (2020) 34:7160–77. 10.1096/fj.201902326R 32259357 PMC7254553

[266] Snider EJ Kubelick KP Tweed K Kim RK Li Y Gao K. Improving stem cell delivery to the trabecular meshwork using magnetic nanoparticles. Sci Rep. (2018) 8:12251. 10.1038/s41598-018-30834-7 30115953 PMC6095892

[267] Snider EJ Vannatta RT Schildmeyer L Stamer WD Ethier CR. Characterizing differences between MSCs and TM cells: Toward autologous stem cell therapies for the glaucomatous trabecular meshwork. J Tissue Eng Regen Med. (2018) 12:695–704. 10.1002/term.2488 28556530

[268] Coulon SJ Schuman JS Du Y Bahrani Fard MR Ethier CR Stamer WD. A novel glaucoma approach: stem cell regeneration of the trabecular meshwork. Prog Retin Eye Res. (2022) 90:101063. 10.1016/j.preteyeres.2022.101063 35398015 PMC9464663

[269] Abu-Hassan DW Li X Ryan EI Acott TS Kelley MJ. Induced pluripotent stem cells restore function in a human cell loss model of open-angle glaucoma. Stem Cells. (2015) 33:751–61. 10.1002/stem.1885 25377070 PMC4359625

[270] Mead B Tomarev S. Bone marrow-derived mesenchymal stem cells-derived exosomes promote survival of retinal ganglion cells through miRNA-dependent mechanisms. Stem Cells Transl Med. (2017) 6:1273–85. 10.1002/sctm.16-0428 28198592 PMC5442835

[271] Harrell CR Simovic Markovic B Fellabaum C Arsenijevic A Djonov V Arsenijevic N. Therapeutic potential of mesenchymal stem cell-derived exosomes in the treatment of eye diseases. Adv Exp Med Biol. (2018) 1089:47–57. 10.1007/5584_2018_219 29774506

[272] Vilela CAP Messias A Calado RT Siqueira RC Silva MJL Covas DT. Retinal function after intravitreal injection of autologous bone marrow-derived mesenchymal stromal cells in advanced glaucoma. Doc Ophthalmol Adv Ophthalmol. (2021) 143:33–8. 10.1007/s10633-021-09817-z 33469852

[273] Borrás T. The pathway from genes to gene therapy in glaucoma: a review of possibilities for using genes as glaucoma drugs. Asia-Pac J Ophthalmol Phila Pa. (2017) 6:80–93. 10.22608/APO.2016126 PMC6005701 28161916

[274] Rhee J Shih KC. Use of gene therapy in retinal ganglion cell neuroprotection: current concepts and future directions. Biomolecules. (2021) 11:581. 10.3390/biom11040581 33920974 PMC8071340

[275] Daliri K Ljubimov AV Hekmatimoghaddam S. Glaucoma, stem cells, and gene therapy: where are we now? Int J Stem Cells. (2017) 10:119–28. 10.15283/ijsc17029 28844129 PMC5741193

[276] Hellström M Ruitenberg MJ Pollett MA Ehlert EME Twisk J Verhaagen J. Cellular tropism and transduction properties of seven adeno-associated viral vector serotypes in adult retina after intravitreal injection. Gene Ther. (2009) 16:521–32. 10.1038/gt.2008.178 19092858

[277] Mowat FM Gornik KR Dinculescu A Boye SL Hauswirth WW Petersen-Jones SM. Tyrosine capsid-mutant AAV vectors for gene delivery to the canine retina from a subretinal or intravitreal approach. Gene Ther. (2014) 21:96–105. 10.1038/gt.2013.64 24225638 PMC3880610

[278] Ratican SE Osborne A Martin KR. Progress in gene therapy to prevent retinal ganglion cell loss in glaucoma and leber's hereditary optic neuropathy. Neural Plast. (2018) 2018:e7108948. 10.1155/2018/7108948 29853847 PMC5954906

[279] Martineau HM Pyrah IT. Review of the application of RNA interference technology in the pharmaceutical industry. Toxicol Pathol. (2007) 35:327–36. 10.1080/01926230701197107 17455080

[280] Tsai H-C Pietrobon V Peng M Wang S Zhao L Marincola FM. Current strategies employed in the manipulation of gene expression for clinical purposes. J Transl Med. (2022) 20:535. 10.1186/s12967-022-03747-3 36401279 PMC9673226

[281] Wu J Bell OH Copland DA Young A Pooley JR Maswood R. Gene therapy for glaucoma by ciliary body aquaporin 1 disruption using CRISPR-Cas9. Mol Ther. (2020) 28:820–9. 10.1016/j.ymthe.2019.12.012 31981492 PMC7054720

[282] Chern KJ Nettesheim ER Reid CA Li NW Marcoe GJ Lipinski DM. Prostaglandin-based rAAV-mediated glaucoma gene therapy in Brown Norway rats. Commun Biol. (2022) 5:1–14. 10.1038/s42003-022-04134-w 36329259 PMC9633612

[283] Borrás T Buie LK Spiga MG Carabana J. Prevention of nocturnal elevation of intraocular pressure by gene transfer of dominant-negative RhoA in rats. JAMA Ophthalmol. (2015) 133:182–90. 10.1001/jamaophthalmol.2014.4747 25412195 PMC4527865

[284] Tan J Liu G Zhu X Wu Z Wang N Zhou L. Lentiviral vector-mediated expression of Exoenzyme C3 transferase lowers intraocular pressure in monkeys. Mol Ther. (2019) 27:1327–38. 10.1016/j.ymthe.2019.04.021 31129118 PMC6612778

[285] Tan J Wang X Cai S He F Zhang D Li D. C3 transferase-expressing scAAV2 transduces ocular anterior segment tissues and lowers intraocular pressure in mouse and monkey. Mol Ther Methods Clin Dev. (2020) 17:143–55. 10.1016/j.omtm.2019.11.017 31909087 PMC6938898

[286] Borrás T Buie LK Spiga MG. Inducible scAAV2 GREMMP1 lowers IOP long-term in a large animal model for steroid-induced glaucoma gene therapy. Gene Ther. (2016) 23:438–49. 10.1038/gt.2016.14 26855269 PMC4860029

[287] Jain A Zode G Kasetti RB Ran FA Yan W Sharma TP. CRISPR-Cas9–based treatment of myocilin-associated glaucoma. Proc Natl Acad Sci. (2017) 114:11199–204. 10.1073/pnas.1706193114 28973933 PMC5651749

[288] Ciociola EC Fernandez E Kaufmann M Klifto MR. Future directions of glaucoma treatment: emerging gene, neuroprotection, nanomedicine, stem cell, and vascular therapies. Curr Opin Ophthalmol. (2024) 35:89–96. 10.1097/ICU.0000000000001016 37910173

[289] Yao K Yu Y Li F Jin P Deng C Zhang H. Integrative analysis of an lncRNA-associated competing endogenous RNA network in human trabecular meshwork cells under oxidative stress. Mol Med Rep. (2020) 21:1606–14. 10.3892/mmr.2020.10955 32016457 PMC7003033

[290] Aebersold AS Song Z-H. The effects of cannabidiol on aqueous humor outflow and trabecular meshwork cell signaling. Cells. (2022) 11:3006. 10.3390/cells11193006 36230968 PMC9564313

[291] Vahabikashi A Gelman A Dong B Gong L Cha EDK Schimmel M. Increased stiffness and flow resistance of the inner wall of Schlemm's canal in glaucomatous human eyes. Proc Natl Acad Sci U S A. (2019) 116:26555–63. 10.1073/pnas.1911837116 31806762 PMC6936716

[292] Ghanem A Mokbel T Ghanem A Elnokrashy A. Rho-kinase inhibitors as a novel medication for glaucoma treatment – a review of the literature. Egypt J Ophthalmol Mansoura Ophthalmic Cent. (2021) 1:110–20. 10.21608/ejomos.2021.57582.1006

[293] Chitranshi N Rajput R Godinez A Pushpitha K Mirzaei M Basavarajappa D. Neuroserpin gene therapy inhibits retinal ganglion cell apoptosis and promotes functional preservation in glaucoma. Mol Ther J Am Soc Gene Ther. (2023) 31:2056–76. 10.1016/j.ymthe.2023.03.008 36905120 PMC10362384

[294] Dias MS Luo X Ribas VT Petrs-Silva H Koch JC. The role of axonal transport in glaucoma. Int J Mol Sci. (2022) 23:3935. 10.3390/ijms23073935 35409291 PMC8999615

[295] Wójcik-Gryciuk A Gajewska-Wozniak O Kordecka K Boguszewski PM Waleszczyk W Skup M. Neuroprotection of retinal ganglion cells with AAV2-BDNF pretreatment restoring normal TrkB receptor protein levels in glaucoma. Int J Mol Sci. (2020) 21:6262. 10.3390/ijms21176262 32872441 PMC7504711

[296] Osborne A Wang AXZ Tassoni A Widdowson PS Martin KR. Design of a novel gene therapy construct to achieve sustained brain-derived neurotrophic factor signaling in neurons. Hum Gene Ther. (2018) 29:828–41. 10.1089/hum.2017.069 29466871 PMC6066195

[297] Nishijima E Honda S Kitamura Y Namekata K Kimura A Guo X. Vision protection and robust axon regeneration in glaucoma models by membrane-associated Trk receptors. Mol Ther. (2023) 31:810–24. 10.1016/j.ymthe.2022.11.018 36463402 PMC10014229

[298] Shiozawa AL Igarashi T Kobayashi M Nakamoto K Kameya S Fujishita S. Tyrosine triple mutated AAV2-BDNF gene therapy in an inner retinal injury model induced by intravitreal injection of N–methyl-D-aspartate (NMDA). Mol Vis. (2020) 26:409–22. 32565669 PMC7300199

[299] Pease ME Zack DJ Berlinicke C Bloom K Cone F Wang Y. Effect of CNTF on retinal ganglion cell survival in experimental glaucoma. Invest Ophthalmol Vis Sci. (2009) 50:2194–200. 10.1167/iovs.08-3013 19060281 PMC2810634

[300] Shen J Xiao R Bair J Wang F Vandenberghe LH Dartt D. Novel engineered, membrane-localized variants of vascular endothelial growth factor (VEGF) protect retinal ganglion cells: a proof-of-concept study. Cell Death Dis. (2018) 9:1–18. 10.1038/s41419-018-1049-0 30282966 PMC6170416

[301] Donahue RJ Fehrman RL Gustafson JR Nickells RW. BCLXL gene therapy moderates neuropathology in the DBA/2J mouse model of inherited glaucoma. Cell Death Dis. (2021) 12:1–11. 10.1038/s41419-021-04068-x 34376637 PMC8355227

[302] Visuvanathan S Baker AN Lagali PS Coupland SG Miller G Hauswirth WW. XIAP gene therapy effects on retinal ganglion cell structure and function in a mouse model of glaucoma. Gene Ther. (2022) 29:147–56. 10.1038/s41434-021-00281-7 34363035

[303] Krishnan A Fei F Jones A Busto P Marshak-Rothstein A Ksander BR. Overexpression of soluble fas ligand following adeno-associated virus gene therapy prevents retinal ganglion cell death in chronic and acute murine models of glaucoma. J Immunol. (2016) 197:4626–38. 10.4049/jimmunol.1601488 27849168 PMC5136323

[304] Fang F Zhuang P Feng X Liu P Liu D Huang H. NMNAT2 is downregulated in glaucomatous RGCs, and RGC-specific gene therapy rescues neurodegeneration and visual function. Mol Ther. (2022) 30:1421–31. 10.1016/j.ymthe.2022.01.035 35114390 PMC9077370

[305] Hines-Beard J Bond WS Backstrom JR Rex TS. Virus-mediated EpoR76E gene therapy preserves vision in a glaucoma model by modulating neuroinflammation and decreasing oxidative stress. J Neuroinflammation. (2016) 13:39. 10.1186/s12974-016-0499-5 26876380 PMC4753658

[306] Guo X Zhou J Starr C Mohns EJ Li Y Chen EP. Preservation of vision after CaMKII-mediated protection of retinal ganglion cells. Cell. (2021) 184:4299–4314.e12. 10.1016/j.cell.2021.06.031 34297923 PMC8530265

[307] Lani-Louzada R Marra C Dias MS de Araújo VG Abreu CA Ribas VT. Neuroprotective gene therapy by overexpression of the transcription factor MAX in rat models of glaucomatous neurodegeneration. Invest Ophthalmol Vis Sci. (2022) 63:5. 10.1167/iovs.63.2.5 35103748 PMC8819487

[308] Fujita K Nishiguchi KM Shiga Y Nakazawa T. Spatially and temporally regulated NRF2 gene therapy using Mcp-1 promoter in retinal ganglion cell injury. Mol Ther Methods Clin Dev. (2017) 5:130–41. 10.1016/j.omtm.2017.04.003 28480312 PMC5415330

[309] Zeng R Li J Gong H Luo J Li Z Ou Z. Hyperbranched cationic glycogen derivative-mediated IκBα gene silencing regulates the uveoscleral outflow pathway in rats. BioMed Res Int. (2020) 2020:e8206849. 10.1155/2020/8206849 33381584 PMC7762656

[310] Abbasi M Gupta VK Chitranshi N Gupta V Ranjbaran R Rajput R. Inner retinal injury in experimental glaucoma is prevented upon AAV mediated Shp2 silencing in a caveolin dependent manner. Theranostics. (2021) 11:6154–72. 10.7150/thno.55472 33995651 PMC8120201

[311] Zhou R-R Li H-B You Q-S Rong R You M-L Xiong K. Silencing of GAS5 alleviates glaucoma in rat models by reducing retinal ganglion cell apoptosis. Hum Gene Ther. (2019) 30:1505–19. 10.1089/hum.2019.056 31608710

[312] Cui Z Kang J Hu D Zhou J Wang Y. Oncomodulin/truncated protamine-mediated Nogo-66 receptor small interference RNA delivery promotes axon regeneration in retinal ganglion cells. Mol Cells. (2014) 37:613–9. 10.14348/molcells.2014.0155 25134537 PMC4145373

[313] Balendra SI Zollet P Cisa Asinari Di Gresy E Casasca G Cordeiro MF. Personalized approaches for the management of glaucoma. Expert Rev Precis Med Drug Dev. (2020) 5:145–64. 10.1080/23808993.2020.1756770

[314] Donthula G Daigavane S. Secondary glaucoma following corneal transplantation: a comprehensive review of pathophysiology and therapeutic approaches. Cureus. (2024) 16:e69882. 10.7759/cureus.69882 39439658 PMC11495823

[315] Souzeau E Goldberg I Healey PR Mills RA Landers J Graham SL. Australian and New Zealand Registry of Advanced Glaucoma: methodology and recruitment. Clin Experiment Ophthalmol. (2012) 40:569–75. 10.1111/j.1442-9071.2011.02742.x 22171965

[316] Cissé Y Bai L Meng T. LncRNAs in genetic basis of glaucoma. BMJ Open Ophthalmol. (2018) 3:131. 10.1136/bmjophth-2017-000131 29963644 PMC6020790

[317] Li F Zhang X. The role of polygenic risk scores in glaucoma management. JAMA Ophthalmol. (2024) 142:220. 10.1001/jamaophthalmol.2024.0220 38483371

[318] Pinazo-Durán MD Zanón-Moreno V García–Villanueva C Martucci A Peris-Martínez C Vila-Arteaga J. Biochemical–molecular–genetic biomarkers in the tear film, aqueous humor, and blood of primary open-angle glaucoma patients. Front Med. (2023) 10:1157773. 10.3389/fmed.2023.1157773 37305138 PMC10251746

[319] Dammak A Sanchez Naves J Huete-Toral F Carracedo G. New biomarker combination related to oxidative stress and inflammation in primary open-angle glaucoma. Life. (2023) 13:1455. 10.3390/life13071455 37511830 PMC10381240

[320] Igarashi N Honjo M Asaoka R Kurano M Yatomi Y Igarashi K. Aqueous autotaxin and TGF-βs are promising diagnostic biomarkers for distinguishing open-angle glaucoma subtypes. Sci Rep. (2021) 11:1408. 10.1038/s41598-021-81048-3 33446826 PMC7809106

[321] Nagstrup AH. The use of benzalkonium chloride in topical glaucoma treatment: an investigation of the efficacy and safety of benzalkonium chloride-preservedintraocular pressure-lowering eye drops and their effect on conjunctival goblet cells. Acta Ophthalmol (Copenh). (2023) 101:3–21. 10.1111/aos.15808 38037546

[322] Lambuk L Suhaimi NAA Sadikan MZ Jafri AJA Ahmad S Nasir NAA. Nanoparticles for the treatment of glaucoma-associated neuroinflammation. Eye Vis. (2022) 9:26. 10.1186/s40662-022-00298-y 35778750 PMC9250254

[323] Almasieh M Wilson AM Morquette B Cueva Vargas JL Di Polo A. The molecular basis of retinal ganglion cell death in glaucoma. Prog Retin Eye Res. (2012) 31:152–81. 10.1016/j.preteyeres.2011.11.002 22155051

